# Sak and Sak4 recombinases are required for bacteriophage replication in *Staphylococcus aureus*

**DOI:** 10.1093/nar/gkx308

**Published:** 2017-05-05

**Authors:** Maan M. Neamah, Ignacio Mir-Sanchis, María López-Sanz, Sonia Acosta, Ignacio Baquedano, Andreas F. Haag, Alberto Marina, Silvia Ayora, José R. Penadés

**Affiliations:** 1 Institute of Infection, Immunity and Inflammation, College of Medical, Veterinary and Life Sciences, University of Glasgow, Glasgow G12 8TA, UK; 2 Department of Microbiology, Faculty of Veterinary Medicine, University of Kufa, Kufa, Iraq; 3 Departamento de Ciencias Biomédicas, Universidad CEU Cardenal Herrera, 46113 Moncada, Valencia, Spain; 4 Department of Microbial Biotechnology, Centro Nacional de Biotecnología, CNB-CSIC, 28049 Madrid, Spain; 5 Instituto de Biomedicina de Valencia (IBV-CSIC) and CIBER de Enfermedades Raras (CIBERER), 46010 Valencia, Spain

## Abstract

DNA-single strand annealing proteins (SSAPs) are recombinases frequently encoded in the genome of many bacteriophages. As SSAPs can promote homologous recombination among DNA substrates with an important degree of divergence, these enzymes are involved both in DNA repair and in the generation of phage mosaicisms. Here, analysing Sak and Sak4 as representatives of two different families of SSAPs present in phages infecting the clinically relevant bacterium *Staphylococcus aureus*, we demonstrate for the first time that these enzymes are absolutely required for phage reproduction. Deletion of the genes encoding these enzymes significantly reduced phage replication and the generation of infectious particles. Complementation studies revealed that these enzymes are required both in the donor (after prophage induction) and in the recipient strain (for infection). Moreover, our results indicated that to perform their function SSAPs require the activity of their cognate single strand binding (Ssb) proteins. Mutational studies demonstrated that the Ssb proteins are also required for phage replication, both in the donor and recipient strain. In summary, our results expand the functions attributed to the Sak and Sak4 proteins, and demonstrate that both SSAPs and Ssb proteins are essential for the life cycle of temperate staphylococcal phages.

## INTRODUCTION

Bacteriophages (phages) are recognised as the most abundant and diverse microorganisms on earth (1). The relevant and versatile roles that phages play in nature is demonstrated in the pathogenic bacterium *Staphylococcus aureus*. In this species, *Siphoviridae* phages are responsible for different human diseases by encoding important toxins, such as exfoliatin A (responsible for the scalded skin syndrome), enterotoxin A (food poisoning) or the Panton-Valentine leukocidin (PVL) (necrotising pneumonia) (2). Phages are also relevant as a major source of genetic variation when *S. aureus* strains from different origins are compared (3). Moreover, horizontal gene transfer in *S. aureus* occurs mainly by phage-mediated transduction (4). A remarkable example of the role of staphylococcal phages in spreading virulence genes is observed with a family of pathogenicity islands, the *S. aureus* pathogenicity islands (SaPIs), which hijack staphylococcal phage machinery to ensure their promiscuous intra- and inter-generic transfer (recently reviewed in (4)). Since these elements encode factors involved in virulence and host adaptation, phages are considered to be the major evolutionary driving force in this species (5). In spite of their relevance, the biology of staphylococcal phages remains poorly understood.

One group of proteins widespread in bacteriophages are the single strand annealing proteins (SSAPs), a subtype of recombinases which has gained attention in recent years not only because of its use in recombineering systems (6) but also because of its impact in the remodeling of bacterial genomes (7). For simplicity we will refer hereafter to SSAP as SSAP or just recombinases (not to be confused with site-specific recombinases coded by temperate bacteriophages that catalyze integration/excision). Different recombinase families have been identified in prophage genomes from both Gram-positive and Gram-negative bacteria. These include Sak, Redβ, Erf, Sak4 and Gp2.5 (8). Sak, Redβ and Erf belong to the Rad52-like superfamily of recombinases, Sak4 belongs to the unrelated Rad51/RecA family, while Gp2.5 defines the third superfamily of these recombinase proteins. In spite of their promiscuous presence in phage genomes, very few members of the different recombinase families have been characterized in detail. Biochemical studies have shown that all the phage recombinases studied so far are SSAPs that can promote genetic recombination under more permissive conditions than RecA (6,9,10). However, their roles *in vivo* have not been studied to any great extent. It has long been assumed that these enzymes play a pivotal role in promoting gene shuffling among temperate phages, accelerating phage evolution (10). One study using *Bacillus subtilis* phage SPP1 suggested that the recombinase G*35*P (Redβ family) could be involved in the generation of the concatemeric DNA during phage replication (11), whereas another study implicated the phage P22-encoded Erf recombinase in the circularization of the phage genome after infection (12). However, for most members of these families their role in the phage cycle remains to be determined.

Remarkably, staphylococcal temperate phages always encode one of these five aforementioned families of SSAPs. Interestingly, although not related in sequence, the genes encoding the SSAP proteins always share the same location in the phage genomes (Supplementary Figure S1) (8,13), suggesting that all have the same role in the phage cycle. Further analysis indicates the constant presence of a gene coding for a predicted protein, homologous to a single strand DNA binding protein (Ssb), adjacent to these recombinase genes (Supplementary Figure S1). Similar to the SSAP proteins, the role of the Ssb proteins in the staphylococcal phage cycle remains to be established. With this in mind, we have explored here the role that different families of SSAPs and their cognate Ssb proteins play in the life cycle of the staphylococcal *Siphoviridae* temperate phages. Our results clearly demonstrate that both staphylococcal phage encoded proteins, SSAP and Ssb, play an essential role in phage replication.

## MATERIALS AND METHODS

### Bacterial strains and growth conditions

The bacterial strains used in this study are listed in Supplementary Table S1. The procedures for preparation and analysis of phage lysates, in addition to transduction and transformation of *S. aureus*, were performed as previously described (14,15).

### DNA methods

General DNA manipulations were performed using standard procedures. The plasmids and oligonucleotides used in this study are listed in Supplementary Tables S2 and S3, respectively. The labeling of probes and DNA hybridizations were performed according to the protocol supplied with the PCR-DIG DNA-labeling and Chemiluminescent Detection Kit (Roche). To produce the phage mutations, we used plasmid pMAD (16), as previously described (17).

### Complementation of the mutants

The different phage genes under study were PCR amplified using oligonucleotides listed in Supplementary Table S3. PCR products were cloned into pCN51 (18) and the resulting plasmids (Supplementary Table S2) were introduced into the appropriate recipient strains (Supplementary Table S1).

### Analysis of phage DNA replication by pulsed field gel electrophoresis (PFGE)

Bacteria were grown in TSB to OD_540_ = 0.3, then the wild-type or prophage mutants under study were induced by Mitomycin C (MC; 2 μg/ml). Cultures were grown at 32°C with slow shaking (80 rpm). Samples (1 ml) were removed at various time-points after phage induction and cells were harvested for 5 min at 12 000 rpm, 4°C. Cells were washed with 1 ml of ice cold washing buffer (Tris 20 mM pH 8.5, 100 mM EDTA, 100 mM NaCl), pelleted and stored at –20°C. Cells were resuspended in 50 μl of buffer P1 (50 mM Tris pH 8.0 10 mM EDTA, 0.1 mg/ml RNAse A) and lysed with lysostaphin (0.2 mg/ml) and lysozyme (0.5 mg/ml) for 30 min at 22°C. Then 50 μl of a solution containing proteinase K (1 mg/ml) plus 2% sodium dodecyl sulphate (SDS) was added and samples were further incubated for 30 min at 55°C. 10 μl aliquots were analysed by agarose gel (0.8%) electrophoresis in Tris-acetate buffer or by PFGE followed by Southern blot.

PFGE was performed with a Bio-Rad CHEF-DR II apparatus. Running conditions were 5 V/cm, 0.5× TBE, 0.5–10 switch time for 20 h at 12°C. The molecular weight marker used was the LW range PFG marker (New England Biolabs). The probe used for Southern blot development was a PCR product of 500-bp complementary to the cI (80α) and Orf15 (ϕ11) gene regions. Southern blots were performed with Hybond-N+ membranes as instructed by the manufacturer (GE Healthcare) and detection was performed with the AlkPhos Direct Labeling kit (GE Healthcare).

### Real-time quantitative PCR

Whole DNA of *S. aureus* strains RN4220, RN10359, JP6001, JP6016, JP6017, JP1361, JP4001, JP4012 and JP4013 was extracted using the GenElute Bacterial Genomic DNA Kit (Sigma-Aldrich) according to the manufacturer's instructions. Real-time quantitative PCR was performed in triplicate using 1 μl DNA as template and Fast SYBR Green Master Mix (Thermofisher Scientific) according to the manufacturer's instructions. Samples were normalized using the levels of *gyr*B and the levels of reconstituted empty attachment sites were calculated relative to the RN4220 control. Primers used for quantitative PCR are listed in Supplementary Table S3. To monitor specificity, the final PCR products were analysed by melting curves and electrophoresis. The relative DNA levels within distinct experiments were determined by using the 2^−ΔΔCT^ method (19). The results show the average ± SEM of at least three independent experiments.

### Protein expression and purification

The 80α *sak* gene was PCR amplified and cloned into NcoI and BamHI cleaved pET-15b plasmid to generate plasmid pET-*sak*. The correct insertion of *sak* into plasmid pET-*sak* was confirmed by sequencing. *Escherichia coli* BL21 (DE3) pLysS cells were transformed with pET-*sak* and protein expression was induced by addition of 0.2 mM IPTG to cells growing in LB at 18°C at an optical density of 0.2 at 560 nm. These were then further incubated for 16 h at 18°C. Cells were collected by centrifugation and stored at –20°C.

Ten grams of wet cells were resuspended in 50 ml of buffer A (50 mM Tris–HCl pH 7.5, 15% glycerol, 1 mM EDTA, 1 mM DTT) containing 300 mM NaCl and lysed by sonication. Cell debris and insoluble proteins were then removed by centrifugation at 18 000 rpm for 45 min at 4°C. Sak protein was precipitated from the soluble fraction by 50% ammonium sulfate saturation, followed by centrifugation at 18 000 rpm for 30 min at 4°C. The pellet was washed once with 50% ammonium sulfate saturation and centrifuged again. The ammonium sulfate pellet containing Sak was resuspended in buffer A containing 500 mM NaCl and was loaded into a 5 ml hydroxyapatite column (Bio-Rad). The column was washed stepwise with increasing phosphate concentrations in buffer A containing 500 mM NaCl. Pure Sak protein was eluted from this column at 200 mM phosphate. Fractions containing pure protein were pooled and precipitated with 70% ammonium sulfate to concentrate the protein. The pellet was solubilized in buffer A containing 500 mM NaCl and 50% glycerol and purified protein was stored at –20°C.

### DNA substrates

Oligo 100-nt-up with the sequence: 5΄- GGGCGAATTGGGCCCGACGTCGCATGCTCCTCTAGACTCGAGGAATTCGGTACCCCGGGTTCGAAATCGATAAGCTTACAGTCTCCATTTAAAGGACAAG-3΄ and its complementary, oligo 100-nt-down, were purchased from SIGMA and purified by PAGE as described before (20).

Oligonucleotide 100-nt-up was radiolabeled at the 5΄-end by polynucleotide kinase and γ^32^P-ATP and was the source of single strand DNA (ssDNA). Its double strand (dsDNA) control was obtained by heating to 95°C for 5 min an equimolar amount of each oligonucleotide in annealing buffer (100 mM sodium phosphate buffer pH 7.5), and then cooling the sample to room temperature over 2 h. A pUC18 HindIII–NdeI 216 bp dsDNA fragment was the source of dsDNA. It was radiolabeled using α^32^P-dATP incorporated using Klenow enzyme.

### Single-strand annealing and binding assays

Binding of Sak to DNA was analysed through electrophoretic mobility-shift assays (EMSA), using ssDNA and dsDNA substrates (0.5 nM). Reactions were performed in buffer B (25 mM Tris–HCl pH 7.5, 0.50 mg/ml BSA, 100 mM NaCl, 5% glycerol, 2 mM DTT) with 1 mM MgCl_2_ for 15 min at 37°C. Complexes were separated by PAGE in 6% polyacrylamide gels run in TBE, and the gels were dried before autoradiography.

The single-strand annealing activity of the Sak protein was analyzed using the two complementary synthetic oligonucleotides (oligo 100-nt-up and oligo 100-nt-down) of which oligo 100-nt-up was radiolabeled. In time-course experiments, a reaction mix was prepared containing 40 nM Sak in buffer B containing 5 mM magnesium acetate and 0.5 nM oligo 100-nt-up. Reactions were initiated by the addition of 0.5 nM cold 100-nt-down DNA. Tubes were placed at 30°C and 10 μl aliquots were removed at times 2, 4, 8, 6, 8, 10 and 12 min. Samples were deproteinized by the addition of one volume of stop buffer (40 mM Tris–HCl pH 7.5, 0.2% SDS, 100 mM EDTA, 0.4 mg/ml proteinase K) followed by a 15 min incubation at 37°C. A control reaction was performed in parallel without protein to observe the spontaneous annealing of the two oligonucleotides under the conditions used. DNA products were analysed by 8% PAGE, run in TBE and autoradiography.

## RESULTS

### The 80α encoded Sak recombinase is required for phage replication

Phage 80α ORF16 (accession number ABF71587) is well conserved among many *S. aureus* phages. Interestingly, the primary sequence of the ORF16 encoded protein shows 42% identity in a stretch of 150 amino acids to the lactococcal phage p2 Sak3 protein (accession number AAR14300; Supplementary Figure S2), and therefore we renamed ORF16 as Sak. Lactococcal Sak3 has been characterised as a single strand-annealing protein (SSAP) belonging to the Sak family of recombinases (8,9). Although the biochemical characterization of Sak3 clearly demonstrated that this protein promotes homologous recombination (9), its role in the phage cycle remains unclear.

**Figure 1.**
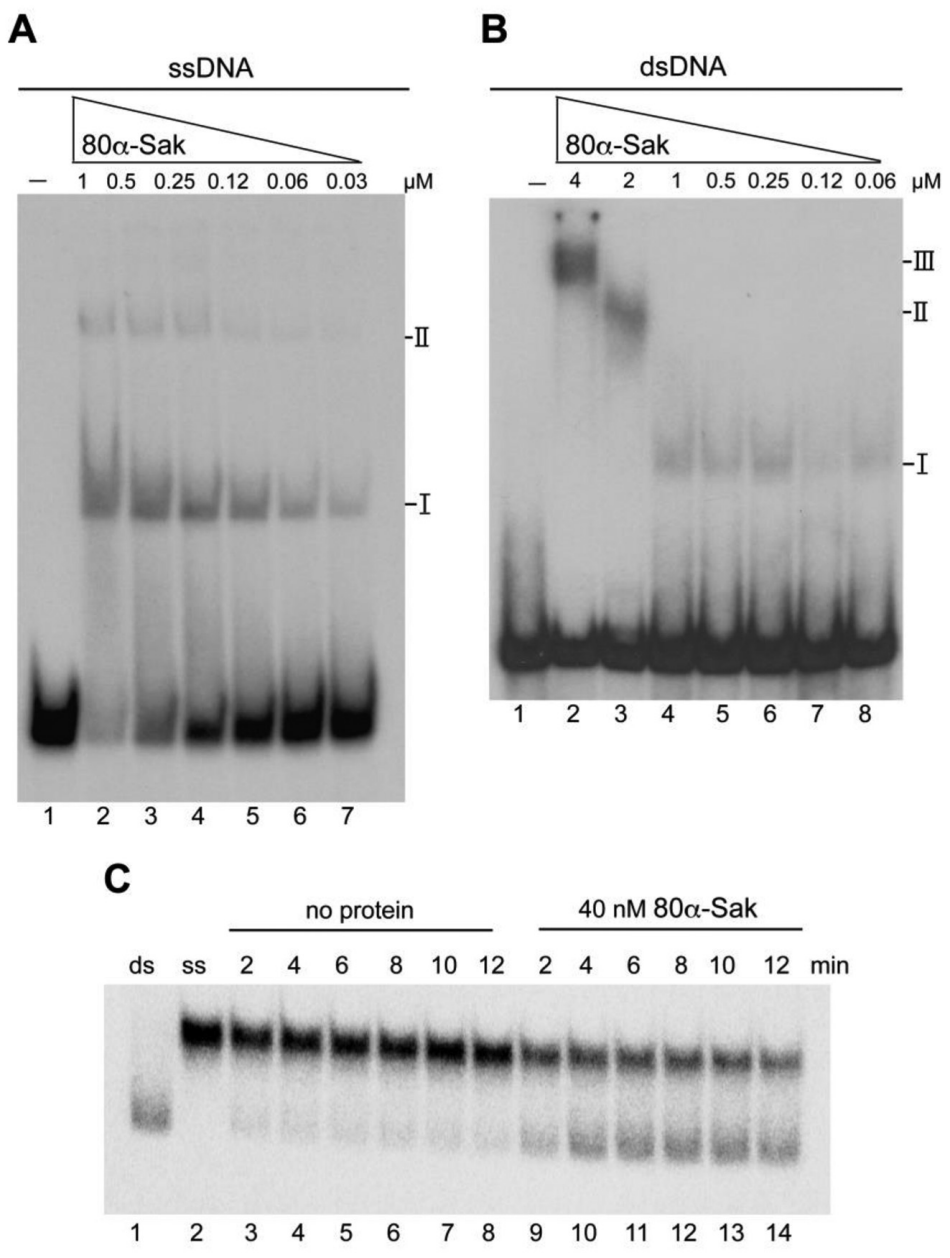
ssDNA binding and annealing activities of *S. aureus* phage 80α Sak protein. (**A**) Electrophoretic mobility shift assay showing the binding of Sak protein to ssDNA. Radiolabelled oligo 100-nt-up (0.5 nM) was incubated with increasing amounts of Sak protein (from 30 nM to 1 μM) in buffer B containing 1 mM MgCl_2_ for 15 min at 37°C. The two types of complexes formed are denoted by I and II. (**B**) Binding to dsDNA. Radiolabelled pUC18 HindIII–NdeI 216 bp dsDNA fragment (0.5 nM) was incubated with increasing amounts of Sak protein (from 60 nM to 4 μM) in buffer B containing 1 mM MgCl_2_ for 15 min at 37°C. The three types of complexes formed are denoted by I, II and III. (**C**) Sak promotes single-strand annealing. Radiolabelled oligo 100-nt-up and its complementary (0.5 nM) were incubated with a fixed concentration of Sak (40 nM) in the presence of 5 mM magnesium acetate. At the given time points, aliquots were removed and deproteinized. The reaction products were analyzed by 8% PAGE and autoradiopgraphy. Lane 1: control annealed 100 bp ds DNA; lane 2: radiolabelled oligo 100-nt-up; lanes 3–8: control reaction without protein (2–12 min); lanes 9–14: reaction with Sak of phage 80α (40 nM, 2–12 min).

We initially analyzed whether the 80α-encoded Sak protein had a similar ability to that of the lactococcal Sak recombinases in its ability to preferentially bind to ssDNA and anneal two complementary ssDNA sequences. We purified the protein and first performed DNA binding studies. As shown in Figure 1A, experiments performed increasing the Sak concentration and keeping the ssDNA concentration constant confirmed that Sak forms stable complexes with ssDNA. The affinity (*K*_app_) for this interaction was ∼200 nM, which is similar to that previously reported for lactococcal Sak3 (9). Similar experiments were performed with dsDNA, but the amount of dsDNA retarded was reduced and the *K*_app_ was >4 μM (Figure 1B). Next, since the 80α-encoded Sak does not contain any Walker motif in its sequence (Supplementary Figure S2), we tested whether Sak can anneal complementary 100-mer oligonucleotides in the absence of ATP. This was the case, and a time-course experiment revealed that Sak annealed complementary oligonucleotides with similar kinetics to Sak3 (Figure 1C) (9). Taken together, these results confirm that the 80α-encoded Sak recombinase is a *bona fide* SSAP.

Next, we introduced an in-frame deletion in this gene in an 80α lysogen and evaluated how this mutation influenced the phage cycle. Surprisingly, this deletion severely affected phage reproduction and phage-mediated bacterial lysis. Thus, cellular lysis after mitomycin C (MC) induction of the 80α *sak* mutant prophage occurred overnight, in clear contrast to the cells carrying the wt 80α prophage which lysed 4 h after MC induction. Moreover, the analysis of the different phage lysates showed that the 80α mutant did not generate plaques in the recipient strain. Complementation of the recipient strain with a pCN51 derivative plasmid expressing the *sak* gene under the control of the *P*cad promoter showed that the 80α mutant was able to generate a few functional phage particles in the donor strain in the absence of the *sak* gene (Table 1). Complementation of the donor but not the recipient strain showed that *sak* is also absolutely required in the recipient strain to form plaques (Table 1). In summary, our complementation studies demonstrated that the effect observed in the 80α mutant depends on the *sak* gene, with this gene being required both in the donor and the recipient strains (Table 1).

**Table 1.** Effect of mutations on phage titer^a^

	Recipient strain		
Donor lysogen	RN4220	RN4220 pCN51-*sak*	RN4220 pCN51-*ssb*^80α^
80α	1.3 × 10^10^	ND^b^	ND
80α Δ*sak*	<10	5.2 × 10^4^	ND
80α Δ*sak* pCN51-*sak*	<10	4.3 × 10^8^	ND
80α Δ*ssb*	1.2 × 10^8, c^	ND	1.3 × 10^8, d^
80α Δ*ssb* pCN51-*ssb*^80α^	3.2 × 10^9, c^	ND	7.2 × 10^9, d^
**Donor lysogen**	**RN4220**	**RN4220 pCN51-*sak4***	**RN4220 pCN51-*ssb*** ^ϕ11^
ϕ11	6.7 × 10^8^	ND	ND
ϕ11 Δ*sak*4	<10	3.2 × 10^3^	ND
ϕ11Δ*sak*4 pCN51-*sak*4	<10	8.2 × 10^8^	ND
ϕ11Δ*ssb*	1.2 × 10^6, c^	ND	1.3 × 10^6, d^
ϕ11Δ*ssb* pCN51-*ssb*^ϕ11^	1.2 × 10^8, c^	ND	5.2 × 10^8, d^

^a^Pfu/ml of induced culture.

^b^ND: Not determined.

^c^Small plaques.

^d^Normal sized plaques.

**Figure 2.**
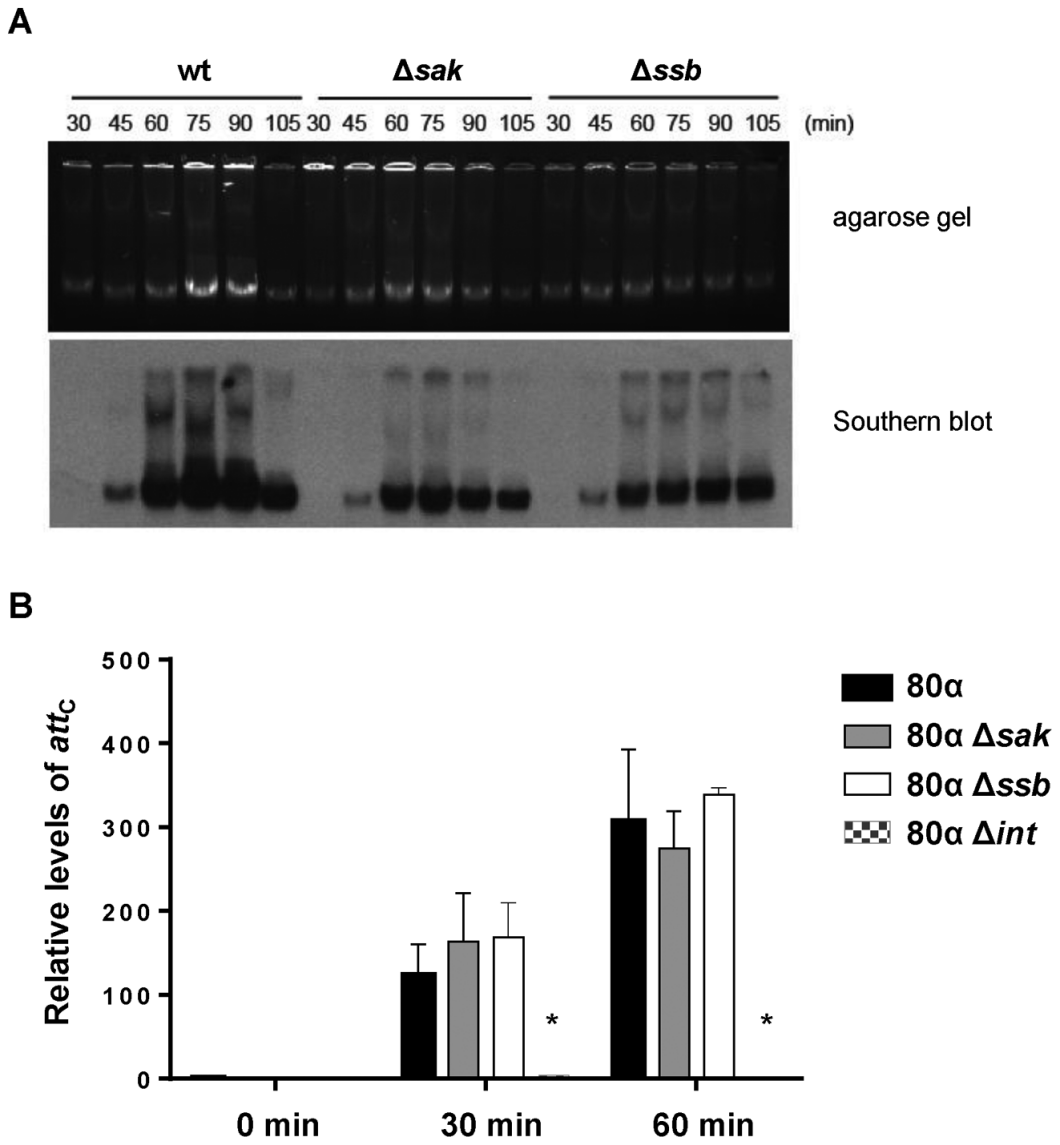
Replication of the 80α *sak* and *ssb* mutants. (**A**) Time course of bacteriophage DNA replication after MC induction. Samples of 80α wt, Δ*sak* and Δ*ssb* mutants were taken at the indicated times after MC induction and separated on agarose. An ethidium bromide stained gel and its Southern blot with a phage-specific probe are shown. (**B**) The 80α Sak and Ssb proteins are not involved in prophage induction. Excision of the 80α wt, Δ*sak*, Δ*ssb* and Δ*int* mutant prophages was investigated at different times (0, 30 or 60 min) after MC induction of the phage lytic cycle. Samples were normalized using the levels of *gyrB* (housekeeping gene). Levels of reconstituted empty *att*_C_ sites were calculated relative to the non-lysogenic strain RN4220 used as a control. Error bars represent SEM. A two-way ANOVA with Holm–Sidak multiple comparisons test was performed to compare mean differences between strains. Differences among the phages were not significant, except for the 80α Δ*int* mutant (*P < 0.0001).

To get insights into the mechanism by which Sak influences the phage cycle, we analyzed its role in phage replication. To do this, both the 80α wt and the *sak* mutant prophages were induced by MC addition and Southern blot analyses with samples taken at different time points were performed. As shown in Figure 2A, in accordance with the results shown in Table 1, replication of the *sak* mutant was significantly reduced.

To rule out the possibility that the observed defects in phage replication were due to ineffective prophage induction, we measured by qPCR the excision activities of the prophages 80α and 80α Δ*sak*, using primers hybridising to the regions flanking the region of phage integration. After 80α induction, the phage is excised and the chromosomal *att*_C_ site is reconstituted. As a control for the experiment, we also included the 80α prophage mutant in the integrase gene (*int*), which does not excise (21). As shown in Figure 2B, excision of the 80α Δ*sak* mutant was similar to wt, while no excision was observed in the 80α Δ*int* mutant, confirming the role of Sak in phage replication but not in excision.

The fact that the Sak recombinase was required for phage replication in *S. aureus* was surprising since in the prototypical phage λ the Redβ recombinase is not required for λ phage replication (22). It has been proposed that phage 80α encodes a replication module of the initiator-helicase loader type, similar to that present in phage λ (23). However, this has not been experimentally demonstrated and the sequence homology of ORF20 and ORF21 with λ proteins O and P is very limited (Supplementary Figures S3A and S4A). A Blast analysis showed that ORF20 has 26% sequence identity (Supplementary Figure S3B) with SPP1 G*38*P, a protein that is essential for DNA replication in phage SPP1, and that has two activities: origin-binding (24), and a PriA-like activity (25). ORF21 showed 27% sequence identity with the *E. coli* DnaC protein (Supplementary Figure S4B, (26)), which acts as a loader of the DnaB replicative helicase (27). Therefore, both proteins are expected to be essential for the initiation of DNA replication in phage 80α. To analyze this, since the precise boundaries of *Staphylococcal siphovirus* DNA replication modules are unknown, we individually deleted in the lysogenic strain containing the 80α prophage the ORFs from ORF9 to ORF37. This region comprises the early transcript of phage 80α (28). Note that although ORFs 7 and 8 also belong to the early transcript of phage 80α, these encode the Cro-like (ORF7) and antirepressor (ORF8) proteins, which are not involved in replication, and consequently, are outside the scope of this study. Analogously, ORFs 22 (Sri) and 38 (RinA), which also belong to this region, have been previously analyzed (14,21,28) and since they do not have a role in replication they will not be further characterized here.

Each strain was SOS induced using MC. Total DNA, containing both bacterial and phage DNA, was prepared from each strain after 60 min, separated on agarose, stained and photographed before Southern blotting with a phage-specific probe. Table 2 summarizes the results obtained with the 80α mutants. Interestingly, deletion of ORFs 20 or 21 completely abolished phage replication (Table 2). In accordance with this, these mutant strains did not lyse, and no phage particles were obtained in the supernatant of the induced strains (Table 2). Moreover, while the 80α ΔORF21 could be complemented in trans, the 80α ORF20 could not (JP14567 and JP15037 respectively, Table 2), suggesting that the 80α replication origin (*ori*) is embedded in this gene. These results showed that just ORF20 and ORF21 are strictly required to initiate DNA replication. However, quantification of the amount of DNA synthesized after 60 min MC induction showed that DNA replication was highly affected in the Δ*sak* (ORF16), as previously reported, and in the Δ*ssb* (ORF17) mutant (Table 2 and Figure 2).

**Table 2.** Effect of phage mutations on 80α titer^a^

80α	Donor lysogen	Lysis^b^	Replication^b^	Complementation^c^	Phage titer^d^
**wt**	RN451	+++	+++		2.8 × 10^10^
**Δ9**	JP5262	+++	+++		2.1 × 10^10^
**Δ10**	JP5224	+++	+++		1.7 × 10^10^
**Δ11**	JP5412	+++	+++		2.1 × 10^10^
**Δ12-13**	MS1531	+++	+++		1.1 × 10^10^
**Δ14**	JP6014	+++	+++		2.1 × 10^10^
**Δ15**	JP6015	+++	+++		2.8 × 10^10^
**Δ16 (*sak*)**	JP6016	+	+	See Table 1	<10
**Δ17 (*ssb*)**	JP6017	+++	++	See Table 1	4.5 × 10^8^
**Δ18**	JP6018	+++	+++		1.7 × 10^10^
**Δ19**	JP6019	+++	+++		1.7 × 10^10^
**Δ20**	JP6020	-	0	-	< 10
	JP15037	-	0	+	< 10
**Δ21**	JP6021	-	0	-	< 10
	JP14567	+++	++	+	1.7 × 10^7^
*Δ22	JP6022	+++	+++		9.8 × 10^9^
**Δ23**	JP6023	+++	+++		1.8 × 10^10^
**Δ24**	JP6024	+++	+++		1.5 × 10^10^
**Δ25**	JP6025	+++	+++		8.2 × 10^9^
**Δ26**	JP6026	+++	+++		1.0 × 10^10^
**Δ27**	JP6027	+++	+++		1.7 × 10^10^
**Δ28**	JP6028	+++	+++		1.4 × 10^10^
**Δ29**	JP6029	+++	+++		2.0 × 10^9^
**Δ30**	JP6030	+++	+++		3.0 × 10^10^
**Δ31**	JP6031	+++	+++		1.0 × 10^10^
**Δ32**	JP4480	+++	+++		2.5 × 10^10^
**Δ33**	JP6033	+++	+++		1.8 × 10^10^
**Δ34**	JP5416	+++	+++		2.2 × 10^10^
**Δ35**	JP5417	+++	+++		1.6 × 10^10^
**Δ36**	JP6036	+++	+++		2.2 × 10^10^
**Δ37**	JP6037	+++	+++		2.7 × 10^10^

^a^The means of results from three independent experiments are shown. Variation was within ±5% in all cases.

^b^Phage replication. +++: not affected; ++: reduced; +: severely reduced; 0: abolished.

^c^In this experiment, only those phage mutants that had affected their life cycle were included. For complementation, the empty pCN51 (–) or the pCN51 derivative plasmid expressing the gene under study (+) was introduced both in the donor and recipient strains, and the phage titer was measured in the recipient strain.

^d^PFU/ml induced culture, using RN4220 as recipient strain.

*Previously analyzed in (14).

To gain further insight into the role of these proteins in DNA replication we analysed whether they were sufficient to support replication of a suicide plasmid. Thus, the 80α genes encoding ORFs 20 and 21 were cloned into a suicide plasmid capable of replicating in *E. coli* but not in S. *aureus*, under the control of a Cd-inducible promoter, generating plasmid pJP1900. This plasmid was successfully transferred to *S. aureus* strain RN4220, where it replicated autonomously. The ability of the transformed strain to form colonies also indicated that the plasmid could segregate. As controls for the experiment two derivatives of the aforementioned plasmid carrying mutations in the 80α ORF20 or ORF21 were generated. Neither plasmid generated colonies in *S. aureus* strain RN4220, suggesting both proteins are necessary and sufficient for 80α replication. To rule out that these suicide plasmids, carrying mutations in the ORFs 20 or 21, did not generate colonies in RN4220 because of a defect in the transformation process, we generated the same constructs but using the thermosensitive (*ts*) plasmid vector pCN50, which replicates very poorly at 43°C. We would expect that all three plasmids (wt carrying the ORF20-21 insert; ORF20 mutant or ORF21 mutant) replicate at 30°C because of the activity of the pCN50-encoded *ts* replicon. However, we would expect that at 43°C only the wt plasmid expressing the ORFs 20 and 21 would support autonomous replication of the pCN50 plasmid. This was the case, and following plating at 43°C, only the strain carrying the wt plasmid expressing the complete 80α replicating module generated colonies. In summary, on the basis of these results we conclude that the 80α ORFs 20–21 complex can drive autonomous replication and segregation of a plasmid in *S. aureus*.

### The single-stranded binding protein Ssb is also required for phage replication

In addition to the aforementioned ORFs, 16 (Sak), 20 and 21, deletion of ORF17 affected DNA replication and phage titre, although to a minor extent (Figure 2 and Table 2). As occurred with the *sak* mutant, the 80α ORF17 mutant did not show any defect in excision after prophage induction (Figure 2B). As previously mentioned, ORF17 encodes a single-strand binding protein (Ssb, Supplementary Figure S5), which is located next to the *sak* gene in all the analyzed phages (Supplementary Figure S1). The fact that the deletion of the phage coded *ssb* gene affects phage reproduction is surprising since 80α Ssb protein shows a high degree of identity with one of the host coded Ssb proteins, the SsbA protein (29). The sequence identity between these two proteins is 79% (Supplementary Figure S5). In SPP1 phage, the phage coded Ssb (G*36*P protein) can be replaced by host SsbA since both proteins have a high degree of sequence identity (25). This putative replacement could partially explain why although most of the plaques generated by this mutant were reduced in size, some plaques were normal (Supplementary Figure S6). To further explore this, we performed complementation studies that demonstrated that expression of the *ssb* gene in the donor strain partially restored the phage titre. The *ssb* gene is required in the recipient strain to fully recover the phage titre and to generate normal sized plaques (Table 1). Thus, the *ssb* gene, as the *sak* gene, is required both in the donor and the recipient strains (Table 1).

To analyze how Ssb affects phage DNA replication, the *ssb* mutant prophage was SOS induced and Southern blot analysis with samples taken at different time points was performed. Similar DNA replication kinetics were observed in the *sak* and *ssb* mutants (Figure 2 and Table 1), confirming that both proteins, Sak and Ssb, are required for 80α replication.

### Sak and Ssb are involved in concatemer formation

**Figure 3.**
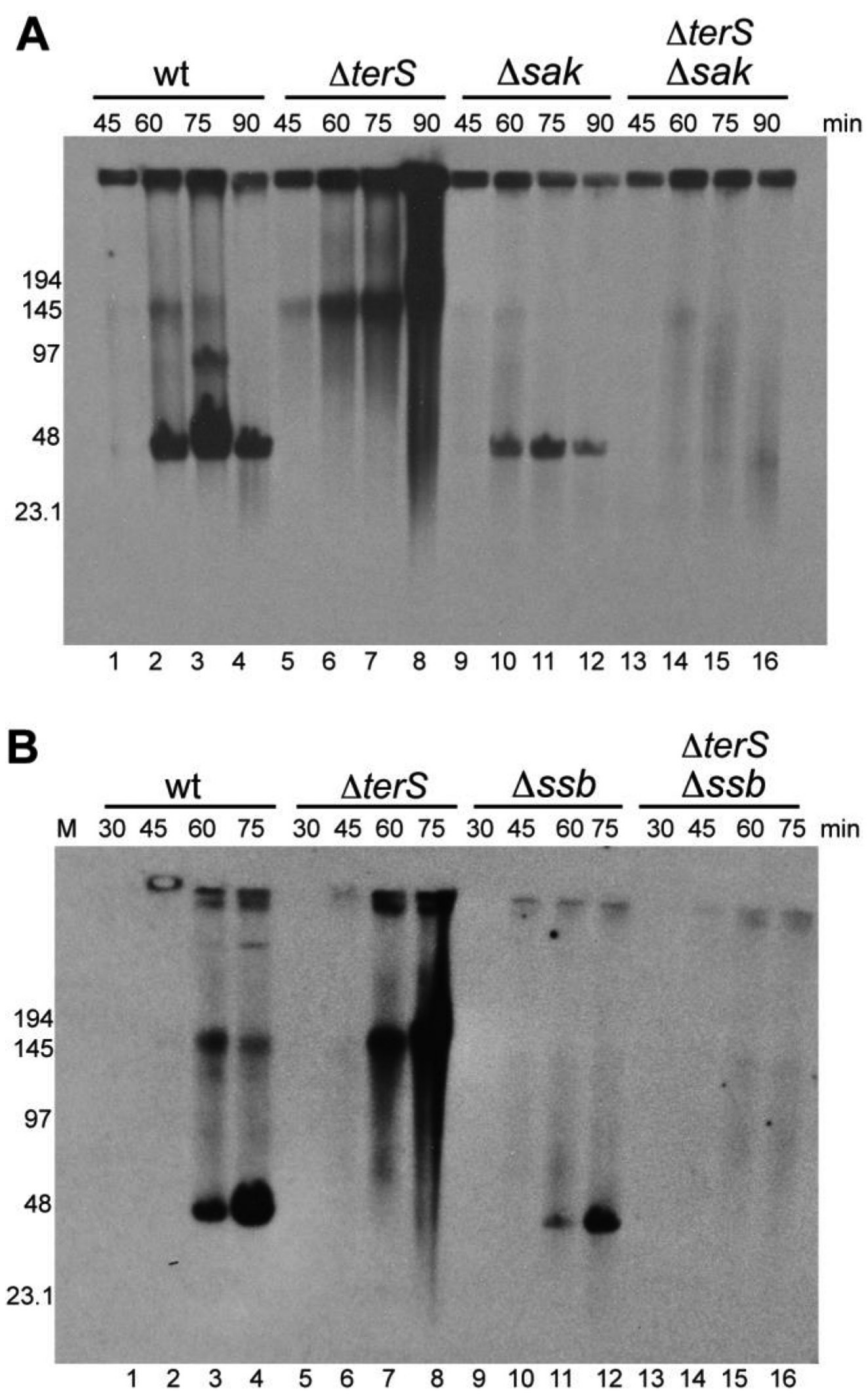
PFGE analysis of 80α replication intermediates. Southern blot showing the time course of bacteriophage DNA replication after MC induction of Δ*sak*, Δ*terS*, and Δ*sak* Δ*terS* mutants (**A**) or after MC induction of Δ*ssb*, Δ*terS*, and Δ*ssb* Δ*terS* mutants (**B**). M is the Low Range PFG Marker. The size of the bands is stated in kilobases.

There are two modes of bacteriophage λ DNA replication during its lytic development in *E. coli* cells. The circle-to-circle (theta; θ) replication predominates at early stages of the phage growth, and depends on the aforementioned O and P proteins (23,30). The rolling-circle (sigma; σ) replication occurs late after infection to produce long concatemers that serve as substrates for packaging of unit-length λ DNA into phage proheads (31). Similarly, in bacteriophage SPP1 DNA replication starts by binding of G*38*P to one of the origins of replication and proceeds by a θ mechanism, before it is shifted later to σ replication (32). The σ mode of DNA replication is essential for concatemer production, which are packaged into the empty proheads by the headful mechanism (33). Phage 80α also packages its concatemeric DNA by a headful mechanism. It has been proposed that in bacteriophage SPP1 the G*35*P recombinase (Redβ family) could be essential for the shift from θ to σ replication (11). Based on the fact that the phage titres observed for both the *sak* and the *ssb* mutants were severely affected compared with phage replication, our hypothesis was that the Sak recombinase and the Ssb protein may be required for the transition from θ to σ replication or for concatemer production in phage 80α. To test this, we analyzed the presence of the concatemeric form in the 80α wt and the *sak* and *ssb* mutant phages. To facilitate this study, we deleted the small terminase gene (*ter*S) both in the 80α wt and the *sak* and *ssb* mutant. Note that TerS is absolutely required for phage DNA packaging (31,34). Consequently, the *ter*S mutant is unable to use the concatemeric form as a substrate for packaging, facilitating the analyses of the presence of this replicating form in the wt and mutant phages. As expected, phage replication was affected in the *sak* Δ*ter*S and *ssb* Δ*ter*S double mutants (Figure 3). However, it was still possible to observe the concatemeric form in these mutants, although to a lesser extent. A possible explanation for this result involving the cellular RecA protein is discussed below.

**Figure 4.**
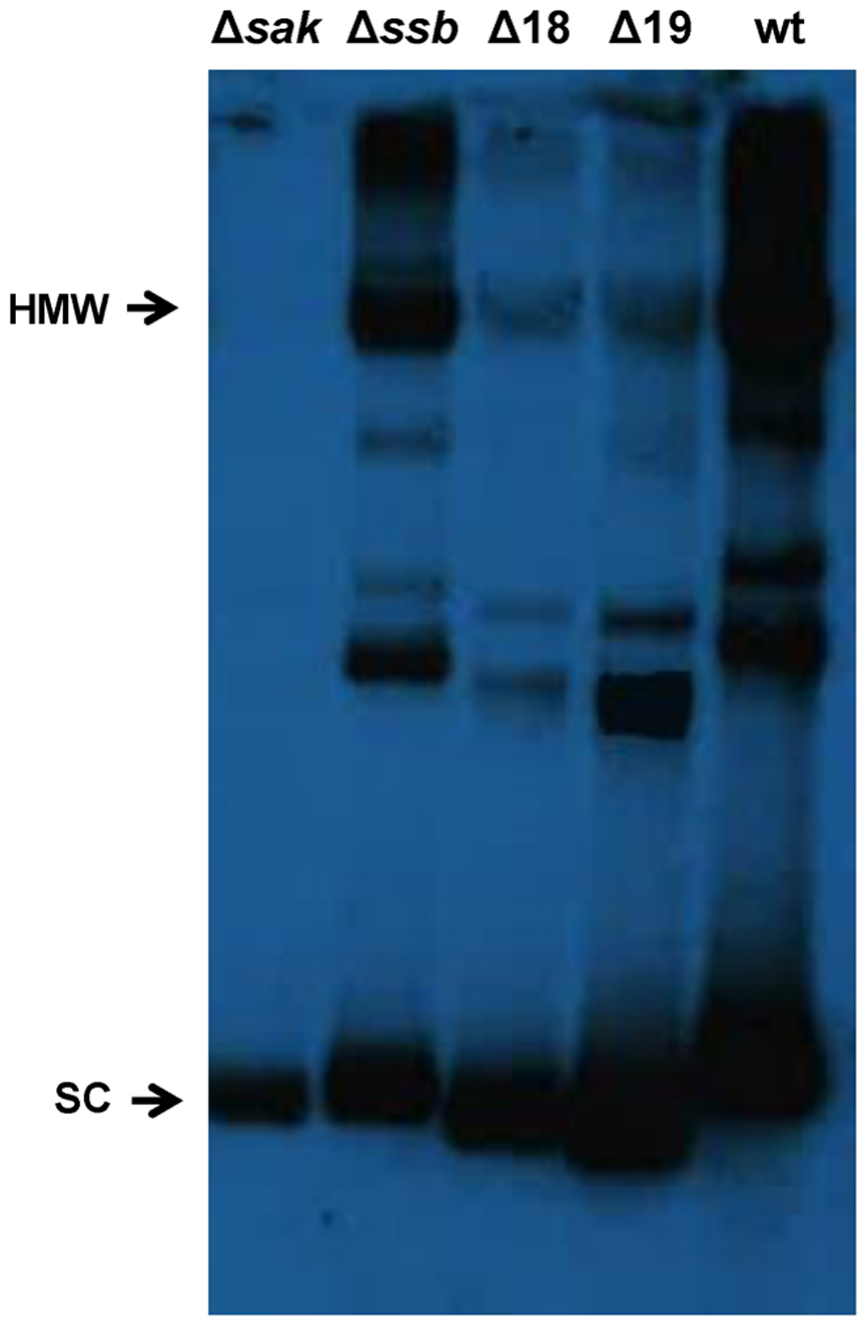
Replication products of 80α subclones. Total DNA from bacterial cultures carrying plasmid pJP1901 (lane 5, designated as wt), or derivatives mutant in the different Orfs (Δ*sak* to Δ19) was extracted and separated by agarose gel electrophoresis, then Southern blotted using a plasmid-specific probe. SC: supercoiled monomers; HMW: high-molecular weight DNA (multimers).

As a complementary strategy, we cloned the PCR product containing ORFs 16–21 under the control of a Cd-inducible promoter, into the aforementioned suicide plasmid. This generated plasmid pJP1901, which expressed Sak (ORF16), Ssb (ORF17) the G*38*P- and DnaC-like proteins (ORFs 20 and 21) plus ORFs 18 and ORF19. As controls we generated a set of derivative suicide plasmids carrying single mutations in each of the aforementioned ORFs. Since expression of ORFs 20 and 21 are sufficient to support θ replication, and since our hypothesis is that both Sak and Ssb can be involved in σ replication, the rationale of the experiment was that DNA replication, or at least the accumulation of high molecular weight DNA (HMW), would be increased in the wt plasmid compared with that present in the *sak* and *ssb* mutants. As expected, plasmid mutants in ORFs 20 and 21 were incapable of replicating autonomously in *S. aureus*. Interestingly, plasmids that could be maintained in *S. aureus* generated different replicating species, some of them HMW, compatible with σ replication, the exception being the plasmid bearing the mutation in the *sak* gene, in which replication was clearly reduced (clearly involving this protein in phage replication). With this plasmid predominantly supercoiled monomeric forms were formed (Figure 4), which are compatible with θ replication. We were unable to see any effect for the *ssb* mutation in these analyses, compatible with its less relevant role in phage replication.

### The unrelated Sak4 recombinase is also required for phage replication

**Figure 5.**
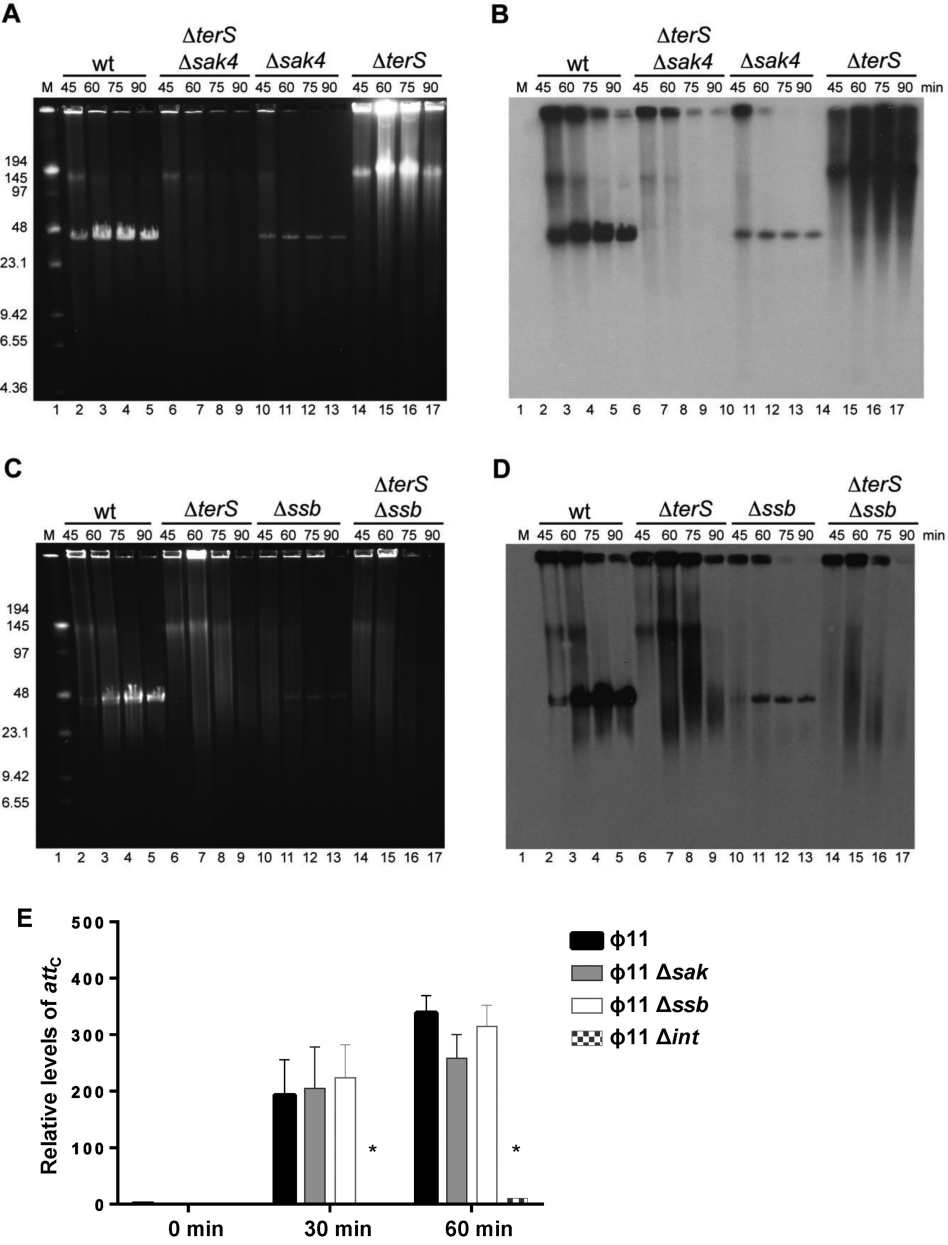
PFGE analysis of DNA replication in ϕ11 mutants. Ethidium bromide gel (**A**) and Southern blot (**B**) showing the time course of bacteriophage DNA replication after MC induction of phage ϕ11 Δ*sak4*, Δ*terS, and* Δ*sak4* Δ*terS* mutants. In (**C**), and (**D**), an ethidium bromide gel and Southern blot showing the time course of bacteriophage DNA replication after MC induction of Δ*ssb*, Δ*terS* and Δ*ssb* Δ*terS* mutants. M, is the Low Range PFG Marker. The size of the bands is stated in kilobases. (**E**) The ϕ11 Sak4 and Ssb proteins are not involved in prophage induction. Excision of the ϕ11 wt, Δ*sak*, Δ*ssb* and Δ*int* mutant prophages was investigated at different times (0, 30 or 60 min) after MC induction of the phage lytic cycle. Samples were normalized using the levels of *gyrB* (house keeping gene). Levels of reconstituted empty *att*_C_ sites were calculated relative to the non-lysogenic strain RN4220 used as a control. Error bars represent SEM. A two-way ANOVA with Holm–Sidak multiple comparisons test was performed to compare mean differences between strains. Differences among the phages were not significant, except for the 80α Δ*int* mutant (*P < 0.0001).

As previously indicated, at least five different recombinase families can be found in the staphylococcal temperate phages: Sak, Erf, Redβ, Sak4 and Gp2.5 families (8). The Sak4 family is less well studied than other families of recombinases. To date, only two reports exist in the literature about Sak4. The first Sak4 protein described was discovered in a genetic screen that showed that mutations in Sak4 of phage 31 conferred the ability to infect a phage-resistant strain of *Lactococcus lactis* expressing the AbiK protein, as it occurs with mutations in the Sak3 proteins (35). A preliminary study of the Sak4 protein encoded by phage PA73 of *Pseudomonas aeruginosa* showed that this protein has a moderate activity in a single strand recombineering assay in *E. coli* (8). As is the case with the Sak family, the role of Sak4 recombinases in the staphylococcal phage cycles remains unsolved, although this family is widely represented in staphylococcal phages (8). To solve this, we analysed the role of the unrelated recombinase Sak4 (NP_803265; Supplementary Figure S7) in the life cycle of ϕ11. An in-frame mutant of the *sak*4 (ORF12) gene was obtained in the ϕ11 prophage. This mutant and the wt prophage were SOS induced, and the phage cycle analysed. As reported for the Sak protein, the Sak4 recombinase was essential for phage replication and transfer (Figure 5 and Table 1), but not for excision of the induced prophage (Figure 5). Complementation studies also confirmed that Sak4 was required both in the donor and recipient cells (Table 1).

### The recombinase and Ssb proteins form a functional cluster involved in phage replication

As previously indicated, in the staphylococcal prophage genomes the *sak* and *ssb* genes cluster together, and this also occurred in prophages with *sak*4 instead of *sak* (Supplementary Figure S1). Since the recombinase-Ssb pair was present in all of the phages analyzed, we investigated whether they comprise a functional unit. First we analysed whether the different recombinases cluster with cognate Ssb proteins, or instead, if there is no association among these proteins. To do this, we randomly selected 14 phages, 7 encoding Sak and 7 encoding Sak4 proteins (see Supplementary Table S4 for details). Supplementary Figure S8A and B shows a comparison among the different phage coded SSAP proteins. Next, we analysed the Ssb proteins encoded by these phages (Supplementary Table S4). As shown in Supplementary Figure S9A, there were two different variants of the Ssb protein: one was present in all the phages encoding Sak (Supplementary Figure S9B), and the other present in phages encoding Sak4 (Supplementary Figure S9C). Thus, the analyses of the different recombinase genes showed that each one is genetically linked with a specific cognate *ssb* gene, suggesting co-ordinated evolution.

To test whether the cognate recombinase-Ssb pairs work co-ordinately, we analysed whether the expression (both in the donor and recipient strains) of the ϕ11 *sak*4 gene could complement the 80α *sak* mutant. Similarly, we investigated whether the expression of the 80α *sak* gene could complement the ϕ11 *sak*4 mutant. Note, as is shown in Supplementary Figure S7, the 80α Sak and the ϕ11 Sak4 proteins are unrelated in sequence. As shown in Table 3, while the ϕ11 Sak4 did not restore the functionality of the phage 80α *sak* mutant, expression of the 80α *sak* gene complemented the ϕ11 *sak*4 mutant. In order to explain this result we hypothesised that the 80α Ssb protein is unable to interact with the ϕ11 Sak4 protein, while the ϕ11 Ssb can interact somehow with the 80α Sak protein. If our hypothesis was true, we would expect that: *i*) the ϕ11 *ssb* gene has a role in phage replication, as previously reported for the 80α *ssb* gene; *ii*) the 80α *ssb* mutant could be complemented with the ϕ11 *ssb* but not vice versa; and *iii*) complementation of the 80α *sak* mutant with a plasmid expressing both the ϕ11 Sak4 and Ssb proteins will restore the phage titre.

**Table 3.** Cross-complementation of the *sak* and *sak*4 mutants^a^

	Recipient strain		
Donor lysogen	RN4220	RN4220 pCN51-*sak*	RN4220 pCN51-*sak4*
80α	8.5 × 10^9^	—^b^	—
80α Δ*sak*	<10	—	—
80α Δ*sak* pCN51-*sak*	—	2.1 × 10^9^	—
80α Δ*sak* pCN51-*sak4*	—	—	<10
ϕ11	6.4 × 10^8^	—	—
ϕ11 Δ*sak4*	<10	—	—
ϕ11Δ*sak4* pCN51-*sak*	—	2.3 × 10^8^	—
ϕ11Δ*sak4* pCN51-*sak4*	—	—	1.1 × 10^8^

^a^Pfu/ml of induced culture.

^b^—: Not determined.

To solve these questions, we deleted the *ssb* gene (ORF13) in the ϕ11 prophage. As occurred with the 80α Ssb, the ϕ11 Ssb is required for phage replication (Figure 5). Moreover, in this mutant both the ϕ11 phage titre and the size of the plaques were significantly affected (Table 1, Supplementary Figure S6). In support of our hypothesis, while expression of the ϕ11*ssb* gene complemented both the 80α and ϕ11 *ssb* mutants, expression of the 80α *ssb* only complemented the cognate 80α *ssb* mutant (Table 4). Finally, while the 80α *sak* mutant cannot be complemented with the ϕ11 *sak4* gene, expression of both the ϕ11 Sak4 and Ssb proteins in the 80α *sak* mutant restored the phage titre (Table 5). These results confirmed the idea that both the phage coded recombinase and Ssb proteins are required for phage replication, working coordinately. In order to know whether the production of concatemeric DNA is affected in ϕ11 *sak4* and *ssb* mutants we combined these mutations with a mutation in the terminase (*ter*S). As occurred in 80α, the formation of concatemeric DNA was reduced, but could still be observed in these mutants (Figure 5).

**Table 4.** Cross-complementation of the *ssb* mutants^a^

	Recipient strain		
Donor lysogen	RN4220	RN4220 pCN51- *ssb*^80α^	RN4220 pCN51- *ssb*^ϕ11^
80α	1.3 × 10^10^	—^b^	—
80α Δ*ssb*	4.0 × 10^7^	—	—
80α Δ*ssb* pCN51-*ssb*^80α^	—	5.1 × 10^9^	—
80α Δ*ssb* pCN51-*ssb*^ϕ11^	—	—	1.0 × 10^9^
ϕ11	6.1 × 10^8^	—	—
ϕ11 Δ*ssb*	1.1 × 10^6^	—	—
ϕ11Δ*ssb* pCN51-*ssb*^80α^	—	1.7 × 10^6^	—
ϕ11Δ*ssb* pCN51-*ssb*^ϕ11^	—	—	2.3 × 10^7^

^a^Pfu/ml of induced culture.

^b^—: Not determined.

**Table 5.** Complementation of the 80α *sak* mutant with the *sak*4-*ssb* pair from ϕ11^a^

	Recipient strain		
Donor lysogen	RN4220	RN4220 pCN51-*sak4*	RN4220 pCN51-*sak4-ssb*^ϕ11^
80α	1.3 × 10^10^	—^b^	—
80α Δ*sak*	<10	—	—
80α Δ*sak* pCN51-*sak4*	—	<10	—
80α Δ*ssb* pCN51-*sak4-ssb*^ϕ11^	—	—	1.8 × 10^8^

^a^Pfu/ml of induced culture.

^b^—: Not determined.

## DISCUSSION

In this work, we have used biochemical and genetic approaches to decipher the roles in DNA replication of genes present in the putative replication operon of staphylococcal phage 80α. Our results show that ORFs 20 and 21 are essential for initiation of DNA replication. ORF20 may be the replisome organizer, bearing in its gene sequence the origin of replication, as it occurs with its homolog the phage SPP1 G*38*P protein (Supplementary Figure S3) (24,36), or with the unrelated lambda O protein (37). In the λ phage, the P protein facilitates replication initiation through recruitment of the *E. coli* replicative helicase DnaB at the *ori*λ by its interaction with the O protein (38,39). Similarly, in phage SPP1, G*38*P binds specifically to the origin of replication (*ori*L) embedded in gene *38* (24,36). In this phage, the helicase and helicase loader are coded by the phage (40) and protein–protein interactions between the phage replicative helicase and the host DnaG primase and the PolIII holoenzyme are essential for recruitment and assembly of a replisome at phage SPP1 *ori*L site (41,42). Since phage 80α does not encode a DNA helicase, and 80α ORF20 and ORF21 are necessary and sufficient to drive autonomous replication in *S. aureus*, the product of the 80α ORF21, a protein homologous to the *E. coli* DnaC-helicase loader (DnaI helicase loader in Gram positives), may be involved in the initiation of DNA replication by recruitment of the host replicative helicase (the DnaC replicative helicase in Gram positives) at the viral origin by protein-protein interaction (43).

We propose that similarly to the λ and SPP1 phages, in phage 80α DNA replication is initiated by the θ mode, and shifts to σ replication, which produces concatemeric DNA. Although extensively studied, the exact mechanism regulating the switch from θ to σ replication in these two phages has not been completely deciphered. In phage λ, host DnaA is considered the main factor involved in the control of the switch from θ- to σ-mode of DNA replication (44–48). In phage SPP1, it is believed that G*38*P tightly bound to the other origin of replication may cause replication stalling and formation of a double strand break. This substrate could be used by the G35P recombinase to re-start DNA replication by the σ mechanism (11). Our mutational analyses showed that mutations in the 80α genes 16 and 17, coding for a recombinase (Sak family) and for an Ssb respectively, affected DNA replication and concatemeric DNA formation. The involvement of recombinases and Ssb proteins in viral DNA replication was further explored in another staphylococcal phage, ϕ11. This phage also codes for a recombinase-Ssb pair although the recombinase belongs to a different protein family, the Sak4 family, and its Ssb has limited homology with the 80α Ssb. The same defect in DNA replication was observed with the ϕ11 mutants. To our knowledge, this study is pioneering in the analyses of the roles of the Sak and Sak4 families of recombinases in DNA replication. We also show for the first time that these proteins work in concert with their cognate Ssb.

Previous work has characterized some recombinases of the Redβ family, and different results were obtained regarding their role in the phage cycle. G*35*P (Redβ family) from phage SPP1 is an essential protein (11). The Redβ recombinase from phage λ is not essential for λ growth in wild-type hosts (49), although λ red mutants grow less well than the wild-type phages (50), and both Redα (a 5΄ to 3΄ exonuclease) and Redβ become essential in *rec*A mutant strains (51). Similarly, *Salmonella* phage P22 *erf* gene is only essential in *rec*A and *rec*J mutant hosts (52,53), and again the P22 *erf* mutant grows less well than wild-type P22 following infection of wild-type host cells (52).

Our results show that not only Sak and Sak4 recombinases, but also their cognate Ssb proteins are required for DNA replication. Interestingly in the lactococcal phage p2, in addition to a Sak recombinase there is a Ssb protein that, as occurs with RecA, interacts with the Sak protein (9), although its role in recombination or *in vivo* has not been analysed. In some coliphages like T4 and T7, phage-encoded Ssbs are essential for DNA replication and cannot be replaced by their host counterparts with which they share no homology (54,55). In contrast, *B. subtilis* phage SPP1 encodes a protein highly homologous to the bacterial-host Ssb (56), and experiments *in vitro* and *in vivo* showed that the bacterial SsbA protein can replace the viral SSB (G*36*P) at the SPP1 replisome (25). Remarkably, our experiments showed that none of the Ssb proteins encoded in the *S. aureus* genome could complement the phages mutant in the *ssb* genes. The genome of *S. aureus* codes for two single-stranded DNA binding proteins called SsbA and SsbB (29), as occurs in other Gram positive bacteria (57) (58). SsbA shows the typical features of the essential bacterial Ssbs, which are homotetramers consisting of four short polypeptides (160–180 aa) with an amphipathic C-terminus involved in the interaction with numerous proteins involved in DNA replication, repair and recombination (59,60). SsbB proteins are non-essential, shorter (130–110 aa), and share identity with the N-terminal DNA-binding domain of SsbA, but lack the characteristic C-terminal region that mediates protein interactions in SsbA (57). The Ssbs encoded by the 80α and ϕ11 phages are very similar to the SsbA protein, and remarkably the C-terminal region is almost identical (Supplementary Figure S5). This homology is even higher between the 80α-Ssb and host SsbA (Supplementary Figure S5). Although the *ssb* mutants are not affected as the *sak* or *sak*4 mutants, the 80α *ssb* mutant shows a 100-fold reduction in the phage titer, while the ϕ11 *ssb* mutant shows a 500-fold reduction in the phage titer. These results suggest that despite its sequence identity, the host SsbA can only partially replace the viral Ssb proteins, which are required for phage replication, at least in the two staphylococcal phages we tested (80α and ϕ11). The fact that the Ssb proteins interact with their cognate SSAPs also suggests that both families of proteins work co-ordinately. Thus, as proposed for the SSAPs proteins, our current hypothesis is that the Ssb proteins could be involved in the transition between the θ and σ replication. This is currently under study.

Although deletion of the *sak* and *sak*4 genes generated a strong defect in phage replication, it was still possible to see in the PFGE analysis some concatemeric forms in the mutant phages. We hypothesize that in these phages, RecA-dependent and RecA-independent pathways of recombination could occur as reported for phage λ (22). In the RecA-independent pathway Sak and Sak4 recombinases may be the main actors in the generation of a concatemer by recombination. In the RecA-dependent pathway, Sak and Sak4 recombinases may act as mediators of RecA, so the concatemeric DNA observed in these mutants may be due to a poorly efficient recombination reaction performed by the host RecA protein in the absence of the SSAPs mediators.

The 80α Sak protein does not have the ATP binding (Walker A) and hydrolysis (Walker B) motifs that are present in many but not all lactococcal Sak proteins (Supplementary Figure S2). Our biochemical analysis showed that 80α Sak protein is able to bind and anneal ssDNA in the absence of ATP. In contrast, the phage p2 Sak3 protein was shown to possess an ATPase activity that is required both for RecA stimulation and DNA binding. None of the staphylococcal Sak proteins that we analyzed possess the ATP binding and hydrolysis motifs (Supplementary Figures S2 and S8). Interestingly, the ATPase domain is widely conserved in the Sak4 superfamily (8). However, mutation of the Walker A motif in the Sak4 recombinase from *Pseudomonas aeruginosa* phage PA73 did not affect its recombination activity, raising questions as to why this domain is required in some but not in all Sak proteins.

What are the roles *in vivo* for the staphylococcal recombinases? Staphylococcal phages have extensive genome mosaicism (61), which, as occurs in other systems, could be attributed to the presence of different recombinases in the phages’ genomes (7). Furthermore, a recent report has shown that different prophage proteins of unknown function activate the host Stk2 kinase, blocking phage infection (62). All the activator proteins described in that report as of unknown functions show a high degree of homology with either the Sak recombinase or the Sak4 protein described in this work, suggesting a novel role for the recombinases. We show in this report that all the recombinases are essential for phage reproduction. Here two modes of recombination, a single-stranded DNA-annealing pathway and a RecA-assisted pathway might take place in the viral replication cycle for the formation of the viral concatemer.

## Supplementary Material

Supplementary Data
