# Supplementary material for: Sak and Sak4 recombinases are required for bacteriophage replication in Staphylococcus aureus
Source: Nucleic Acids Res. 2017 May 5;45(11):6507–19. doi: 10.1093/nar/gkx308 (PMC5499656; doi:10.1093/nar/gkx308)
Supplement: Supplementary Data [file gkx308_Supp.zip › nar-03563-v-2016-File007.pdf]

Supplementary Table S1. Strains used in this study.

Strains	Description	Reference
S. aureus		
RN4220	Restriction-defective derivative of RN450	(1)
RN450	Non-lysogenic strain	(2)
RN10359	RN450 lysogenic for 80α	(3)
JP1361	RN451 ϕ11	(2)
JP4001	RN451 ϕ11 ΔORF1 (*int*)	(4)
JP4012	RN451 ϕ11 ΔORF12	This work
JP4013	RN451 ϕ11 ΔORF13	This work
JP6001	RN10359 80α ΔORF01 (*int*)	(4)
JP6009	RN10359 80α ΔORF09	This work
JP6010	RN10359 80α ΔORF10	This work
JP6011	RN10359 80α ΔORF11	This work
JP6012	RN10359 80α ΔORF12	This work
JP6013	RN10359 80α ΔORF13	This work
JP6014	RN10359 80α ΔORF14	This work
JP6015	RN10359 80α ΔORF15	This work
JP6016	RN10359 80α ΔORF16	This work
JP6017	RN10359 80α ΔORF17	This work
JP6018	RN10359 80α ΔORF18	This work
JP6019	RN10359 80α ΔORF19	This work
JP6020	RN10359 80α ΔORF20	This work
JP6021	RN10359 80α ΔORF21	This work
JP6022	RN10359 80α ΔORF22	This work
JP6023	RN10359 80α ΔORF23	This work
JP6024	RN10359 80α ΔORF24	This work
JP6025	RN10359 80α ΔORF25	This work
JP6026	RN10359 80α ΔORF26	This work
JP6027	RN10359 80α ΔORF27	This work
JP6028	RN10359 80α ΔORF28	This work
JP6029	RN10359 80α ΔORF29	This work
JP6030	RN10359 80α ΔORF30	This work
JP6031	RN10359 80α ΔORF31	This work
JP6032	RN10359 80α ΔORF32	This work
JP6033	RN10359 80α ΔORF33	This work
JP6034	RN10359 80α ΔORF34	This work
JP6035	RN10359 80α ΔORF35	This work
JP6036	RN10359 80α ΔORF36	This work
JP6037	RN10359 80α ΔORF37	This work
JP15025	RN4220 + pCN51	This work
JP15021	RN4220 + pJP1902	This work
JP15022	RN4220 + pJP1903	This work
JP15023	RN4220 + pJP1904	This work

Strains	Description	Reference
JP15024	RN4220 + pJP1905	This work
JP12551	RN4220 + pJP1901	This work
JP14451	RN4220 + pJP1921	This work
JP14452	RN4220 + pJP1922	This work
JP14453	RN4220 + pJP1923	This work
JP12565	RN4220 + pJP1883	This work
JP12554	JP6016 + pJP1883	This work
JP12553	JP6016 + pCN51	This work
JP14569	RN4220 + pJP1884	This work
JP14542	RN4220 + pJP1887	This work
JP14553	JP6016 + pJP1887	This work
JP14539	RN4220 + pJP1886	This work
JP14548	JP4013 + pJP1886	This work
JP14805	JP4013 + pJP1884	This work
JP15026	JP6017 + pJP1884	This work
JP14804	JP6017 + pJP1886	This work
JP15036	RN4220 + pJP1888	This work
JP15037	JP6020 + pJP1888	This work
JP15038	RN4220 + pJP1889	This work
JP14567	JP6021 + pJP1889	This work
JP12871	RN4220 80α Δ*ter*S	(5)
JP3377	RN451 phi11 ΔterS	(6)
JP15030	RN4220 80α Δ*ter*S ΔORF16	This work
JP15031	RN4220 80α Δ*ter*S ΔORF17	This work
JP15032	RN451 ϕ11 Δ*ter*S ΔORF12	This work
JP14558	RN451 ϕ11 Δ*ter*S ΔORF13	This work

Supplementary Table S2. Plasmids used in this study

Plasmids	Description	Reference
pCN51	Expression vector	(7)
tspCN50	Thermosensitive expression vector	This work
pMAD	Vector for efficient allelic replacement	(8)
pJP1883	pCN51 expressing 80α ORF16	This work
pJP1884	pCN51 expressing 80α ORF17	This work
pJP1888	pCN51 expressing 80α ORF20	This work
pJP1889	pCN51 expressing 80α ORF21	This work
pJP1885	pCN51 expressing ϕ11 ORF12	This work
pJP1886	pCN51 expressing ϕ11 ORF13 phi11	This work
pJP1887	pCN51 expressing ϕ11 ORFs 12 and 13	This work
pJP1897	Suicide plasmid in *S. aureus*. pCN51 derivative.	This work
pJP1898	pJP1897 expressing 80α ORF20	This work
pJP1899	pJP1897 expressing 80α ORF20	This work
pJP1900	pJP1897 expressing 80α ORFs 20 and 21	This work
pJP1908	*ts*pCN50 + pCad promoter + 80α ORF20	This work
pJP1909	*ts*pCN50 + pCad promoter + 80α ORF21 phi80α	This work
pJP1910	*ts*pCN50 + pCad promoter + 80α ORFs 20 and 21	This work
pJP1901	pJP1897 expressing 80α ORFs 16-21	This work
pJP1902	pJP1901 ΔORF16	This work
pJP1903	pJP1901 ΔORF17	This work
pJP1904	pJP1901 ΔORF18	This work
pJP1905	pJP1901 ΔORF19	This work
pJP1906	pJP1901 ΔORF20	This work
pJP1907	pJP1901 ΔORF21	This work
pJP1921	*ts*pCN50 +pCad promoter + ORFs 80α 16-21	This work
pJP1922	pJP1921 ΔORF20	This work
pJP1923	pJP1921 ΔORF21	This work
pJP641	pMAD derivative, deletion of ϕ11 ORF12	This work
pJP1961	pMAD derivative, deletion of ϕ11 ORF13	This work
pJP1932	pMAD derivative, deletion of 80α ORF09	This work
pJP1933	pMAD derivative, deletion of 80α ORF10	This work
pJP1934	pMAD derivative, deletion of 80α ORF11	This work
pJP1935	pMAD derivative, deletion of 80α ORF12	This work
pJP1936	pMAD derivative, deletion of 80α ORF13	This work
pJP1937	pMAD derivative, deletion of 80α ORF14	This work
pJP1938	pMAD derivative, deletion of 80α ORF15	This work
pJP1939	pMAD derivative, deletion of 80α ORF16	This work
pJP1940	pMAD derivative, deletion of 80α ORF17	This work
pJP1941	pMAD derivative, deletion of 80α ORF18	This work
pJP1942	pMAD derivative, deletion of 80α ORF19	This work
pJP1943	pMAD derivative, deletion of 80α ORF20	This work

Plasmids	Description	Reference
pJP1944	pMAD derivative, deletion of 80α ORF21	This work
pJP1945	pMAD derivative, deletion of 80α ORF22	This work
pJP1946	pMAD derivative, deletion of 80α ORF23	This work
pJP1947	pMAD derivative, deletion of 80α ORF24	This work
pJP1948	pMAD derivative, deletion of 80α ORF25	This work
pJP1949	pMAD derivative, deletion of 80α ORF26	This work
pJP1950	pMAD derivative, deletion of 80α ORF27	This work
pJP1951	pMAD derivative, deletion of 80α ORF28	This work
pJP1952	pMAD derivative, deletion of 80α ORF29	This work
pJP1953	pMAD derivative, deletion of 80α ORF30	This work
pJP1954	pMAD derivative, deletion of 80α ORF31	This work
pJP1955	pMAD derivative, deletion of 80α ORF32	This work
pJP1956	pMAD derivative, deletion of 80α ORF33	This work
pJP1957	pMAD derivative, deletion of 80α ORF34	This work
pJP1958	pMAD derivative, deletion of 80α ORF35	This work
pJP1959	pMAD derivative, deletion of 80α ORF36	This work
pJP1960	pMAD derivative, deletion of 80α ORF37	This work

Supplementary Table S3. Oligonucleotides used in this study.

Plasmid	Oligonucleotides	Sequence 5’-3’	DNA template
pJP1883	ORF16 phi80α-21mS	ACGCGTCGACTGATATGTCTAAGCACAAAGC	RN10359
	ORF16 phi80α-20cB	CGCGGATCCTTATTGTTTCTCCTCACTATC	
pJP1901	orf16phi80α-22mNarI	GATAATGGCGCCTGATATGTCTAAGCACAAAGC	RN10359
	Orf21phi80α-6cE	CCGGAATTCGTACATATCTGAACACTCAAG	
pJP1902	orf16phi80α-22mNarI	GATAATGGCGCCTGATATGTCTAAGCACAAAGC	JP6016
	Orf21phi80α-6cE	CCGGAATTCGTACATATCTGAACACTCAAG	
pJP1903	orf16phi80α-22mNarI	GATAATGGCGCCTGATATGTCTAAGCACAAAGC	JP6017
	Orf21phi80α-6cE	CCGGAATTCGTACATATCTGAACACTCAAG	
pJP1904	orf16phi80α-22mNarI	GATAATGGCGCCTGATATGTCTAAGCACAAAGC	JP6018
	Orf21phi80α-6cE	CCGGAATTCGTACATATCTGAACACTCAAG	
pJP1905	orf16phi80α-22mNarI	GATAATGGCGCCTGATATGTCTAAGCACAAAGC	JP6019
	Orf21phi80α-6cE	CCGGAATTCGTACATATCTGAACACTCAAG	
pJP1906	orf16phi80α-22mNarI	GATAATGGCGCCTGATATGTCTAAGCACAAAGC	JP6020
	Orf21phi80α-6cE	CCGGAATTCGTACATATCTGAACACTCAAG	
pJP1907	orf16phi80α-22mNarI	GATAATGGCGCCTGATATGTCTAAGCACAAAGC	JP6021
	Orf21phi80α-6cE	CCGGAATTCGTACATATCTGAACACTCAAG	
pJP1897	pCN33-5mE	CCGGAATTCAATTGAATGAGACATGCTAC	pCN51
	pCad-cadC-5cNarI	GATAATGGCGCCGTTCAGACATTGACCTTCAC	
pJP1888	Orf20phi80α -5mB	CGCGGATCCATCTAACGCAGTAGCGATAAC	RN10359
	Orf20phi80α -6cE	CCGGAATTCAATAGTGGTTTCATAATATCCC	
pJP1889	Orf21phi80α-5mB	CGCGGATCCGGAACGCATGAAGTACGACG	RN10359
	Orf21phi80α-6cE	CCGGAATTCGTACATATCTGAACACTCAAG	
pJP1898	ORF20 phi80α-16mNarI	CATGGCGCCCACCCCCAATCTAACGCAGTAG	RN10359
	Orf20phi80α -6cE	CCGGAATTCAATAGTGGTTTCATAATATCCC	
pJP1899	ORF21 phi80α-16mNarI	CATGGCGCCCACGGATCAATTGGAACGCATG	RN10359
	orf21-phi80α-7cE	CCGGAATTCGGTTACCATGCGTCTCGCTCCC	
pJP1900	ORF20 phi80α-16mNarI	CATGGCGCCCACCCCCAATCTAACGCAGTAG	RN10359
	orf21-phi80α-7cE	CCGGAATTCGGTTACCATGCGTCTCGCTCCC	
pJP1884	ORF17 phi80α-17mS	ACGCGTCGACCAAAAACAAATCGCAGAAGCAC	RN10359
	orf17phi80α-10cB	CGCGGATCCTCAGAACGGTAAGTCATCATC	
pJP1885	ORF12 phi11-1mS	ACGCGTCGACGAAACCAACGTCATACACGG	JP1561
	ORF12 phi11-1cB	CGCGGATCCGTGTTCTACCAGTGATTTTC	
pJP1886	phi-SaPI3-9mS	ACGCGTCGACGTATTAACGATGTTGTACAAGC	JP1561
	orf13-phi11-8cE	CCGGAATTCGTATTGCATTTAAACCACGTC	
pJP1887	ORF13 phi80-2mS	ACGCGTCGACGAGAAACCAACGTCATACAC	JP1561
	orf14-phi11-8cE	CCGGAATTCTTTGCTGTCATCAAACATGCC	
pJP1939	Orf16phi80α-1mB	CGCGGATCCCTATACTTCACTACAGCATGG	RN10359
	Orf16phi80α-2c	CACGTTTTTACTGTTCAACTG	
	Orf16phi80α-3m	CAGTTGAACAGTAAAAACGTGATCGATAAAACGATGAGATGG	
	Orf16phi80α-4cS	ACGCGTCGACATTCGAAACGGATCTATGACG	

Plasmid	Oligonucleotides	Sequence 5'-3'	DNA template
pJP1940	Orf17phi80α-1mB	CGCGGATCCAAACTATTGAGTACGAGGAGG	RN10359
	Orf17phi80α-2c	TGGATCTTTTGTTAAGCGTCC	
	Orf17phi80α-3m	GGACGCTTAACAAAAGATCCAAGTGTTCAATTCTTAGAACCG	
	Orf17phi80α-4cE	CCGGAATTCTGTTGCTCGTTGTGATGTTCG	
pJP1943	Orf20phi80α-1mS	ACGCGTCGACTATTGTTTCTAGGTTGTGTCC	RN10359
	Orf20phi80α-2c	GTGTACTGTGACAAAGTTGCC	
	Orf20phi80α-3m	GGCAACTTTGTCTCACAGTACACGAATGGTTAAACACGGATA GC	
	Orf20phi80α-4cE	CCGGAATTCCTACTCTGTTATCAACAATGC	
pJP1944	Orf21phi80α-1mB	CGCGGATCCCATTCAACTGATGGACTTAGC	RN10359
	Orf21phi80α-2c	ACATCTCTCACATTTCAATCC	
	Orf21phi80α-3m	GGATTGAAATGTGAGAGATGTTTCAGCATTGTTGATAACAGAG	
	Orf21phi80α-4cE	CCGGAATTCTCGACCATGATTTAAGTAATGG	
pJP641	Orf12phi11-1mB	CGCGGATCCCTGTGATGAAGAGACATGACG	JP1361
	Orf12phi11-2c	TTTTTCAGTCATCTATTCTCC	
	Orf12phi11-3m	GGAGAATAGATGACTGAAAAAACAAAGATAAGACACTCAAGC	
	Orf12phi11-4cE	CCGGAATTCGTCCGGAACCTCTACTTCTGC	
pJP1961	Orf13phi11-1mB	CGCGGATCCATAAATGAGGACGGAACAACG	JP1361
	Orf13phi11-2c	TTGAATGTATTGTGTTCTACC	
	Orf13phi11-3m	GGTAGAACACAATACATTCAAATCAATGATGATGATTTACCG	
	Orf13phi11-4cE	CCGGAATTCCTTTCAGCATTTTGTTGAGCC	
pJP1932	Orf9phi80α-1mB	CGCGGATCCGAAATCATGTTGATAGCGAGG	RN10359
	Orf9phi80α-2c	GACTTTTTTGTTTTGAGCTTGC	
	Orf9phi80α-3m	GCAAGCTCAAAACAAAAAAGTCGCAGATATCAAATCAAGGTTT G	
	Orf9phi80α-4cS	ACGCGTCGACCATTTGTTGAATGTGTGGTGG	
pJP1933	Orf10phi80α-1mB	CGCGGATCCGGTAACATCAGATGTCCTACC	RN10359
	Orf10phi80α-2c	TTTAAGAGGTGCATTGCTCGG	
	Orf10phi80α-3m	CCGAGCAATGCACCTCTTAAAGCTCAGAGCAAATCATTGTTG	
	Orf10phi80α-4cS	ACGCGTCGACAATACATCTTGACTTCCTCCG	
pJP1934	orf11phi80α-1mB	CGCGGATCCCGTCTGTGTTCTCTTCAATCC	RN10359
	Orf11phi80α-2c	GGCTTGATTACTAAGTTCACC	
	Orf11phi80α-3m	GGTGAACTTAGTAATCAAGCCGAAAGCTTAAAAGCTCAACAA G	
	Orf11phi80α-4cS	ACGCGTCGACACACGAAGCTAGATTACAACG	
pJP1935 pJP1936	Orf13phi80α-1mB	CGCGGATCCCCTGACTTAGATAATCTGTTCG	RN10359
	Orf13phi80α-2c	CATAATACTGCTAATAGGTAGC	
	Orf13phi80α-3m	CTACCTATTAGCAGTATTATGAGTAACAGTATCAAACACTTAA G	
	Orf13phi80α-4cS	ACGCGTCGACATATCTGGATGTGGAAACTCG	
pJP1937	Orf14phi80α-1mB	CGCGGATCCCTCAGAGCAAATCATTGTTGG	RN10359
	Orf14phi80α-2c	AATACATCTTGACTTCCTCCG	
	Orf14phi80α-3m	CGGAGGAAGTCAAGATGTATTAACCAAGCAATAGATGAATGG	
	Orf14phi80α-4cS	ACGCGTCGACAAGCCTAAACCGTGTAAAGCC	

Plasmid	Oligonucleotides	Sequence 5'-3'	DNA template
pJP1938	Orf15phi80α-1mB	CGCGGATCCTTATCACCTCCTTTCACTAGG	RN10359
	Orf15phi80α-2c	TACAGTCTGCTTCATAGTGAC	
	Orf15phi80α-3m	GTCACTATGAAGCAGACTGTAATGTCTAAGCACAAAGCAATC	
	Orf15phi80α-4cE	CCGGAATTCTATTGTTTCTCCTCACTATCC	
pJP1941	Orf18phi80α-2mB	CGCGGATCCTACCAAGTGCAAGTGACAACG	RN10359
	Orf18phi80α-3c	GATGACAACTGTTGTTGTACC	
	Orf19phi80α-4m	GGTACAACAACAGTTGTCATCTGGATAAAAGTTGATGAGAGG	
	Orf18phi80α-5cE	CCGGAATTCATTTTCTCTTCTAGGTCCTGC	
pJP1942	Orf19phi80α-2mE	CCGGAATTCCCAACCTAAAATCTTGATTCC	RN10359
	Orf19phi80α-3c	ATTTCCCAAAGACAAGTTCCC	
	Orf19phi80α-4m	GGGAACTTGTCTTTGGGAAATAAAGGCTCAATTAGTTTGTCC	
	Orf19phi80α-5cN	CATGCCATGGGCAACGACGAAAAATATTCGC	
pJP1945	Orf22phi80α-1mB	CGCGGATCCAGCAATAGAGTACGTACAAGG	RN10359
	orf22phi80α-2c	GTACATATCTGAACACTCAAG	
	Orf22phi80α-2m	CTTGAGTGTTCAGATATGTACCTTGCAGAACGTCACACACG	
	Orf22phi80α-10cE	CCGGAATTCCTAGTCCATAACGCTTTGAGC	
pJP1946	Orf23phi80α-1mB	CGCGGATCCTATAAGCGAAACAAGCAACGG	RN10359
	Orf23phi80α-2c	TAACACTCCTTAATATTCGACG	
	Orf23phi80α-3m	GTCGAATATTAAGGAGTGTTAGGACTTCTATATGAGCAAGAG	
	Orf23phi80α-4cE	CCGGAATTCATGTAAGGCTTCTAACAACGC	
pJP1947	Orf23phi80α-1mB	CGCGGATCCTATAAGCGAAACAAGCAACGG	RN10359
	Orf24phi80α-1c	CATAGGTGCCTCGAACGTTTC	
	Orf24phi80α-2m	GAAACGTTCGAGGCACCTATGGCATGTAATGGTCATGTGTGG	
	Orf24phi80α-3cE	CCGGAATTCTGCTTCTGTTCCTCGTATTCC	
pJP1948	Orf25phi80α-1mB	CGCGGATCCCTTGCAGAACGTCACACACC	RN10359
	Orf25phi80α-2c	CATTAGATCACTTTCTCAACTC	
	Orf25phi80α-3m	GTTGAGGAAGTGATCTAATGGACGAATTACTAGAGTATGAC	
	Orf25phi80α-4cE	CCGGAATTCTGATAGCTCCGGATTTTCTCG	
pJP1949	Orf25phi80α-1mB	CGCGGATCCCTTGCAGAACGTCACACACC	RN10359
	Orf26phi80α-1c	CACCTTATTTACTCACAGCAC	
	Orf26phi80α-2m	TGGAATAAATGAGTGTCGTGAACCAAATGTTCAAGAAGTGG	
	Orf27phi80α-4cE	CCGGAATTCAAGGCTCCTTCTTTAAACTCG	
pJP1950	Orf27phi80α-1mB	CGCGGATCCAACTAGCTCAAAGCGTTATGG	RN10359
	Orf27phi80α-2c	GGTTTTAGGCATTTATACTTCC	
	Orf27phi80α-3m	GAAGTATAAATGCCTAAAACCTTGGAATACGAGGAACAGAAG	
	Orf27phi80α-4cE	CCGGAATTCAAGGCTCCTTCTTTAAACTCG	
pJP1951	Orf28phi80α-1mB	CGCGGATCCCGTGAAGATTAACGGTAAACC	RN10359
	Orf28phi80α-2c	TGATAGCTCCGGATTTTCTCG	
	Orf28phi80α-3m	CGAGAAAATCCGGAGCTATCATGTTTATATGGCAGATGTGTG	
	Orf28phi80α-4cE	CCGGAATTCACGCTCTTGCAATACCTATCC	
pJP1952	Orf29phi80α-1mB	CGCGGATCCAATGCCTAAAACCGATAACGC	RN10359
	Orf29phi80α-2c	ATCGATTTCGTCAATAATACTC	
	Orf29phi80α-3m	GAGTATTATTGACGAAATCGATGAATTACTAAGTGAAAATGAC G	
	Orf29phi80α-4cS	ACGCGTCGACTTCGAGTACGACAGTTTCAGC	

Plasmid	Oligonucleotides	Sequence 5'-3'	DNA template
pJP1953	Orf30phi80α-1mB	CGCGGATCCTGGATTCGTTCGTTTTCATCC	RN10359
	Orf30phi80α-2c	ATATTGTTCATCTGATAAGGTG	
	Orf30phi80α-3m	CACCTTATCAGATGAACAATATTGGGATAGGTATTGCAAGAGC	
	Orf30phi80α-4cS	ACGCGTCGACTCGATACGGTAACTACCTAGC	
pJP1954	Orf31phi80α-1mB	CGCGGATCCCGTTAATCTGGAAAGATGGGG	RN10359
	Orf31phi80α-2c	GGACTGTTCGATTTTTTCGCG	
	Orf31phi80α-3m	GCGAAAAAATCGAACAGTCCGAACTTGATGAAACAGTAGGG	
	Orf31phi80α-4cS	ACGCGTCGACCTGACCCACTTTAATAACTGC	
pJP1955	Orf32 phi80α-3mB	CGCGGATCCATCGAGTTTAAAGAAGGAGCC	RN10359
	Orf32 phi80α-4c	CGTCTTATGATTTCGTTCGGG	
	Orf32 phi80α-5m	CCCGAACGAAATCATAAGACGCCTGAACTAAAGCAAGTGGAG	
	Orf32 phi80α-6cS	ACGCGTCGACGCATCATTCTTAACATAGCCC	
pJP1956	Orf33phi80α-1mB	CGCGGATCCGTGTATAAGAAAGCGCAAGCG	RN10359
	Orf33phi80α-2c	CGTCACTTCACCAAAACCTCC	
	Orf33phi80α-3m	AGGTTTTGGTGAAGTGACGGCAGTTATTAAAGTGGGTCAG	
	Orf33phi80α-4cS	ACGCGTCGACACCTAGCTTGTATATCTGCGC	
pJP1957	Orf34phi80α-1mB	CGCGGATCCTATTCTCAGCTGAAACTGTCG	RN10359
	Orf34phi80α-2c	AATAGTTTTAATTCTAACCTCCG	
	Orf34phi80α-3m	CGGAGGTTAGAATTAAAACTATTGTTATGAAGTTAGGGATTGAG	
	Orf34phi80α-4cS	ACGCGTCGACTCTTTACTTCGTATAAGACCG	
pJP1958	Orf35phi80α-1mB	CGCGGATCCTGTCGGACTATTAACTAGTCG	RN10359
	Orf35phi80α-2c	CGAGAATTTCATTTTAGGTATG	
	Orf35phi80α-3m	CATACCTAAAATGAAATTCTCGAGTGAATTTTACAGTCCTACG	
	Orf35phi80α-4cS	ACGCGTCGACGCTCTTTAACTTTCACATCGC	
pJP1959	phi80a-7mB	CGCGGATCCAGAGTCAAGGAGGTTTTGGTG	RN10359
	phi80a-8c	GCCGAATAATCGCAGTATTCC	
	phi80a-9m	GGAATACTGCGATTATTCGGCCCGGTCTTATACGAAGTAAAG	
	phi80a-10cS	ACGCGTCGACCTCTTCCCCTAACTCTTCCGC	
pJP1960	phi80a-11mB	CGCGGATCCGAATTTTACAGTCCTACGATGGC	RN10359
	phi80a-12c	TGCTAGTAAGAATAATAGTCTTAG	
	phi80a-13m	CTAAGACTATTATTCTTACTAGCACAGTCAGATTTGATGAGGGCG	
	phi80a-10cS	ACGCGTCGACCTCTTCCCCTAACTCTTCCGC	

qPCR

Oligonucleotides	Sequence 5'-3'	Descrption
Sa-attC-80m	CTGTGGTTCTTACAATGGCG	80α att_{C} site
Sa-attC-80c	CATCGTTGCATTCGTATTACCTAG	
Sa5 attC-11m	GAAAGAAGGAAGTTTAAGACGATG	ϕ11 att_{C} site
Sa-attC-11c	CACAGTGGCTACAACGCATATTAC	
Sa_*gyrB*_+238_F	ACGGATAACGGACGTGGTAT	*gyr*B
Sa_*gyrB*_+339_R	GCCAAATTTACCACCAGCAT	

Supplementary Table S4. SSAP and Ssb proteins analysed in this study.

Phage	Sak	Ssb
80α	ABF71587	ABF71588
53	YP_239694	YP_239695
85	YP_239777	YP_239778
X2	YP_240871	YP_240872
88	YP_240716	YP_240717
phiMR25	YP_001949813	YP_001949814
P954	YP_003169647	YP_003169648
**Phage**	**Sak4**	**Ssb**
11	NP_803265	NP_803266
52A	YP_240651	YP_240652
80	YP_009268656	YP_009268657
96	YP_240275	YP_240276
phiETA	NP_510911	NP_510912
13	NP_803367	NP_803368
phiETA2	YP_001004276	YP_001004277

References

1. Kreiswirth,B.N., Löfdahl,S., Betley,M.J., O'Reilly,M., Schlievert,P.M., Bergdoll,M.S. and Novick,R.P. (1983) The toxic shock syndrome exotoxin structural gene is not detectably transmitted by a prophage. *Nature*, **305**, 709–712.

2. Novick,R. (1967) Properties of a cryptic high-frequency transducing phage in *Staphylococcus aureus*. *Virology*, **33**, 155–166.

3. Ubeda,C., Barry,P., Penadés,J.R. and Novick,R.P. (2007) A pathogenicity island replicon in *Staphylococcus aureus* replicates as an unstable plasmid. *Proc. Natl. Acad. Sci. U.S.A.*, **104**, 14182–14188.

4. Ferrer,M.D., Quiles-Puchalt,N., Harwich,M.D., Tormo-Más,M.Á., Campoy,S., Barbé,J., Lasa,I., Novick,R.P., Christie,G.E. and Penadés,J.R. (2011) RinA controls phage-mediated packaging and transfer of virulence genes in Gram-positive bacteria. *Nucleic Acids Res.*, **39**, 5866–5878.

5. Ubeda,C., Olivarez,N.P., Barry,P., Wang,H., Kong,X., Matthews,A., Tallent,S.M., Christie,G.E. and Novick,R.P. (2009) Specificity of staphylococcal phage and SaPI DNA packaging as revealed by integrase and terminase mutations. *Mol. Microbiol.*, **72**, 98–108.

6. Tormo,M.A., Ferrer,M.D., Maiques,E., Ubeda,C., Selva,L., Lasa,I., Calvete,J.J., Novick,R.P. and Penadés,J.R. (2008) *Staphylococcus aureus* pathogenicity island DNA is packaged in particles composed of phage proteins. *J. Bacteriol.*, **190**, 2434–2440.

7. Charpentier,E., Anton,A.I., Barry,P., Alfonso,B., Fang,Y. and Novick,R.P. (2004) Novel cassette-based shuttle vector system for gram-positive bacteria. *Appl. Environ. Microbiol.*, **70**, 6076–6085.

8. Arnaud,M., Chastanet,A. and Débarbouillé,M. (2004) New vector for efficient allelic replacement in naturally nontransformable, low-GC-content, gram-positive bacteria. *Appl. Environ. Microbiol.*, **70**, 6887–6891.
