# Supplementary material for: Sak and Sak4 recombinases are required for bacteriophage replication in Staphylococcus aureus
Source: Nucleic Acids Res. 2017 May 5;45(11):6507–19. doi: 10.1093/nar/gkx308 (PMC5499656; doi:10.1093/nar/gkx308)
Supplement: Supplementary Data [file gkx308_Supp.zip › nar-03563-v-2016-File008.pdf]

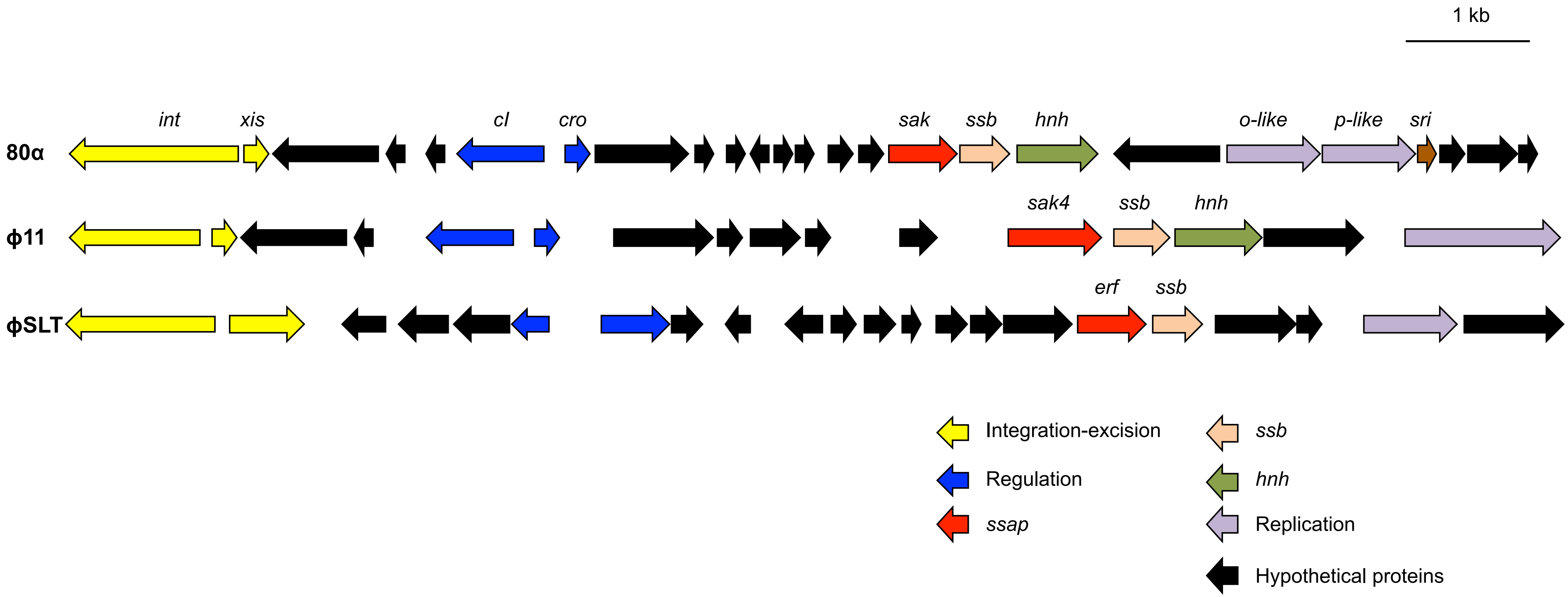

Figure S1. Localisation of the *ssap* and *ssb* genes in the staphylococcal phage genomes. The 5' region from phages 80α, ϕ11 and ϕSLT is shown.

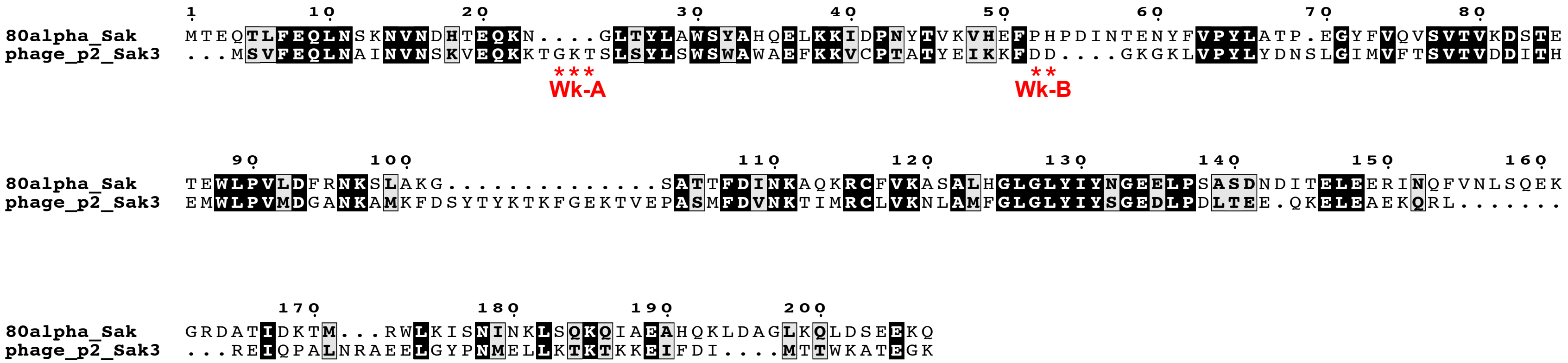

Figure S2. Comparison of the 80α Sak protein with the Sak3 protein of *Lacotoccus lactis* phage p2. Alignment was done with the M-coffee program and depicted with the ESPript server. Identical residues are black boxed. Conserved residues are grey boxed. The red stars identify the ATPase motifs (Walker A and B) present in the Sak3 protein of *L. Lactis* phage p2.

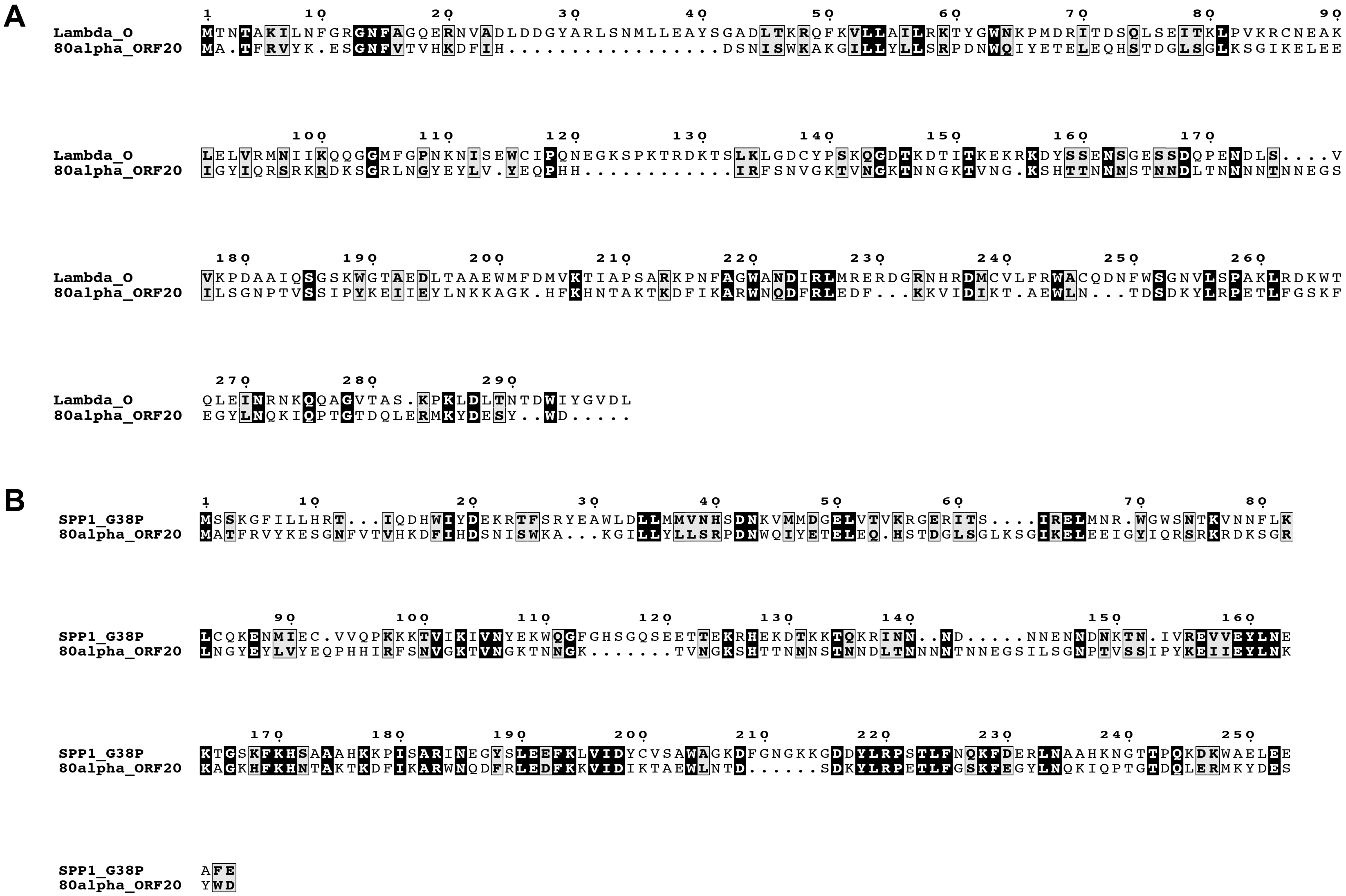

Figure S3. Comparison of the phage λ (A) and phage SPP1 G*38*P proteins (B) with the 80α encoded ORF 20. Identical residues are black boxed. Conserved residues are grey boxed.

A

```
Lambda_P       MKNI.....AAQMVNFDREQMRRI.....ANNMP.......................EQYDEKPQVQQVAQIINGVFSQLLAT...FPAS
80alpha_ORF21  MKPLFSEKINESLKKYQPTHVEKGLKCERCGSEYDLYKFAPTKKHPNGYEYKDGCKCEIYEEYKRN..KQRKINNIFNQSNVNPSLRDAT

Lambda_P       LAN.RDQNEVNEIRRQWVLAFRENGITTMEQVNAGMRVARRQNRPFLPSPGQFVAWCREEASVTAGLPNVSELVDMVYEYCRKRGLYPDA
80alpha_ORF21  VKNYKPQNEKQVHAKQTAIEYVQ.GFSTKEPKSLILQGSYGTGKSHLAY...AIAKAVKAKGHTVAFMHIPMLMDRIKATYNKNAVETTD

Lambda_P       ESYPWKSNAHYWLVTNLYQNMRANALTDAELRRKAADELVHMT.ARINRGEAIPEPVKQLPVMGGRPLNRAQ....AL.AKIAEIKAKFG
80alpha_ORF21  ELVRLLSDIDLLVLDDM...........GVENTEHTLNKLFSIVDNRVGKNNIFTTNFSDKELNQNMNWQRINSRMKHNARKVRVIGDDF.

Lambda_P       LKGAS.V
80alpha_ORF21  .RERDAW
```

B

```
Ecoli_DnaC     MKNVG..DLMQRLQKMMPAHIKPAFKTGE...ELLAWQK...........EQGAIRSAA..LERENRAMKMQRTFNRSGIRPLHQNCSFE
80alpha_ORF21  MKPLFSEKINESLKKYQPTHVEKGLKCERCGSEYDLYKFAPTKKHPNGYEYKDGCKCEIYEEYKRNKQRKINNIFNQSNVNPSLRDATVK

Ecoli_DnaC     NYRVECEGQMNALSKARQYVEEFDGN.IASFIFSGKPGTGKNHLAAAICNELLLRGKSVLIITVADIMSAMKDTF.RNSGTSEEQLLNDL
80alpha_ORF21  NYKPQNEKQVHAKQTAIEYVQGFSTKEPKSLILQGSYGTGKSHLAYAIAKAVKAKGHTVAFMHIPMLMDRIKATYNKNAVETTDELVRLL

Ecoli_DnaC     SNVDLLVIDEIGVQT.ESKYEKVIINQIVDRRSSSKRPTGMLTNSNMEEMTKLLG.ERVMDRMRLGNSLWVIFNWDSYRSRVTGKEY
80alpha_ORF21  SDIDLLVLDDMGVENTEHTLNK.LFS.IVDNRVGKN.N.IFTTNFSDKELNQNMNWQRINSRMK.HNARKVRVIGDDFRER...DAW
```

Figure S4. Comparison of the phage λ P (A) and *E. coli* DnaC proteins (B) with the 80α encoded ORF21. Identical residues are black boxed. Conserved residues are grey boxed.

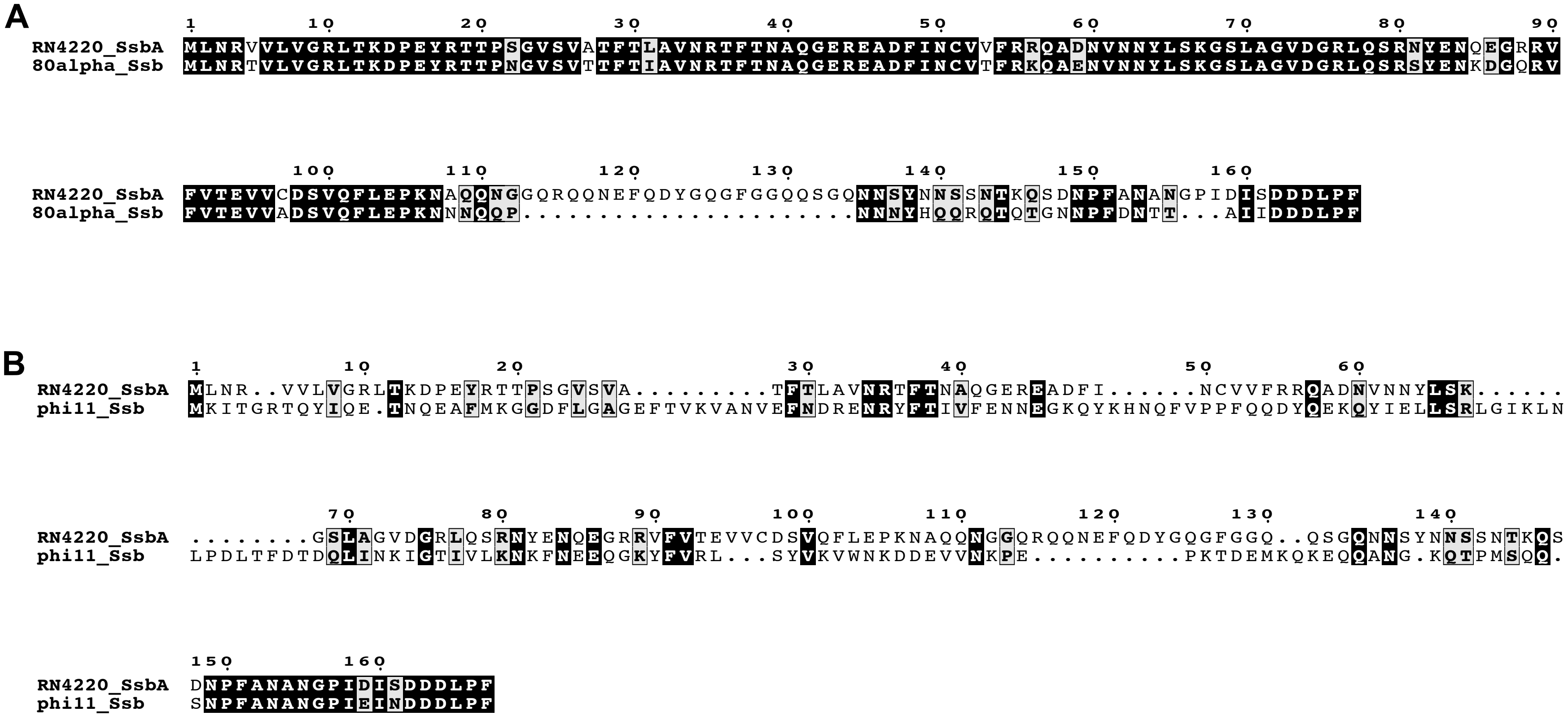

Figure S5. Comparison of the Ssb proteins coded by 80α (A) and ϕ11 (B) phages with the SsbA protein from *S. aureus*. Alignment was done with the M-coffee program and depicted with the ESPript server. Identical residues are black boxed. Conserved residues are grey boxed.

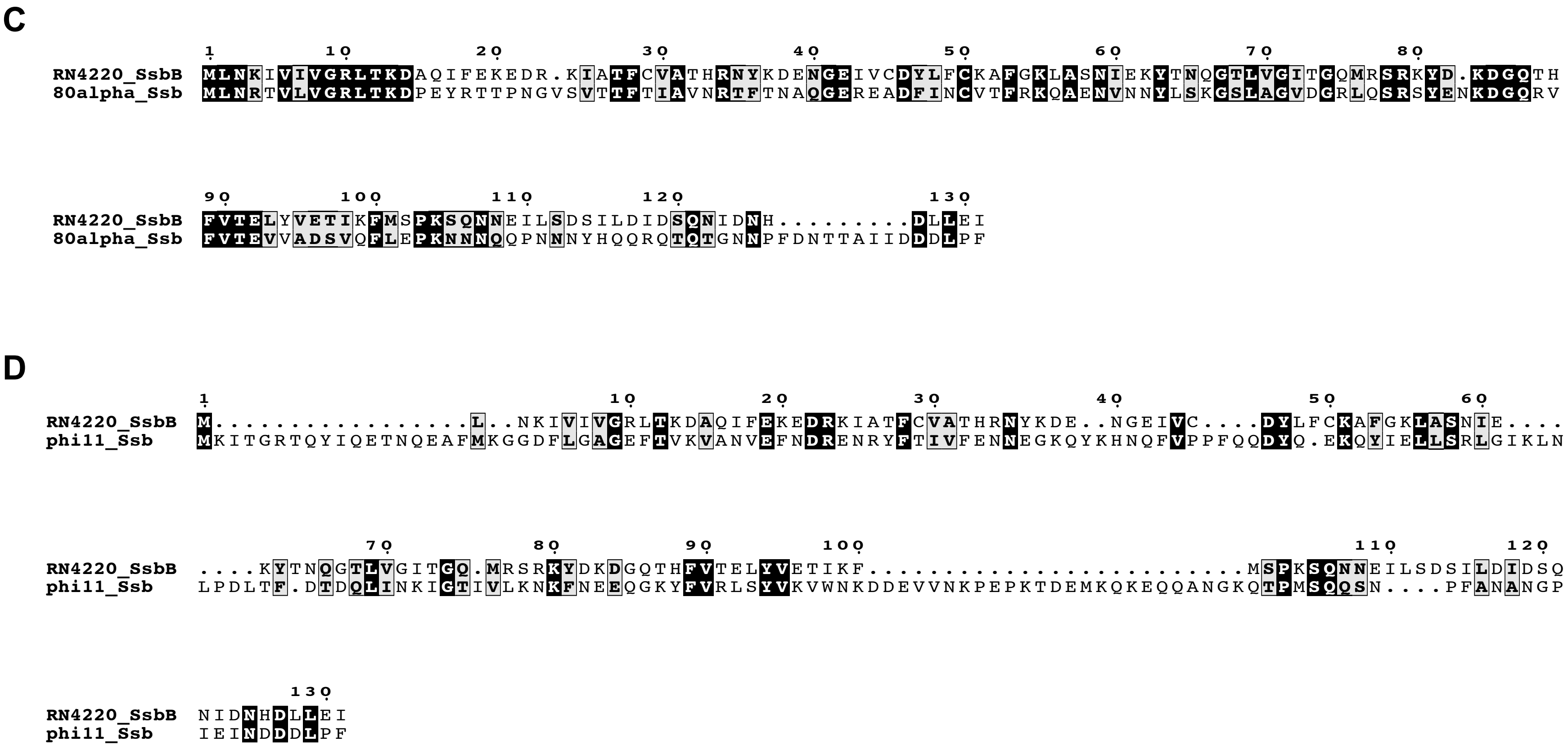

Figure S5 (cont.). Comparison of the Ssb proteins coded by 80α (C) and ϕ11 (D) phages with the SsbB protein from *S. aureus*. Alignment was done with the M-coffee program and depicted with the ESPript server. Identical residues are black boxed. Conserved residues are grey boxed.

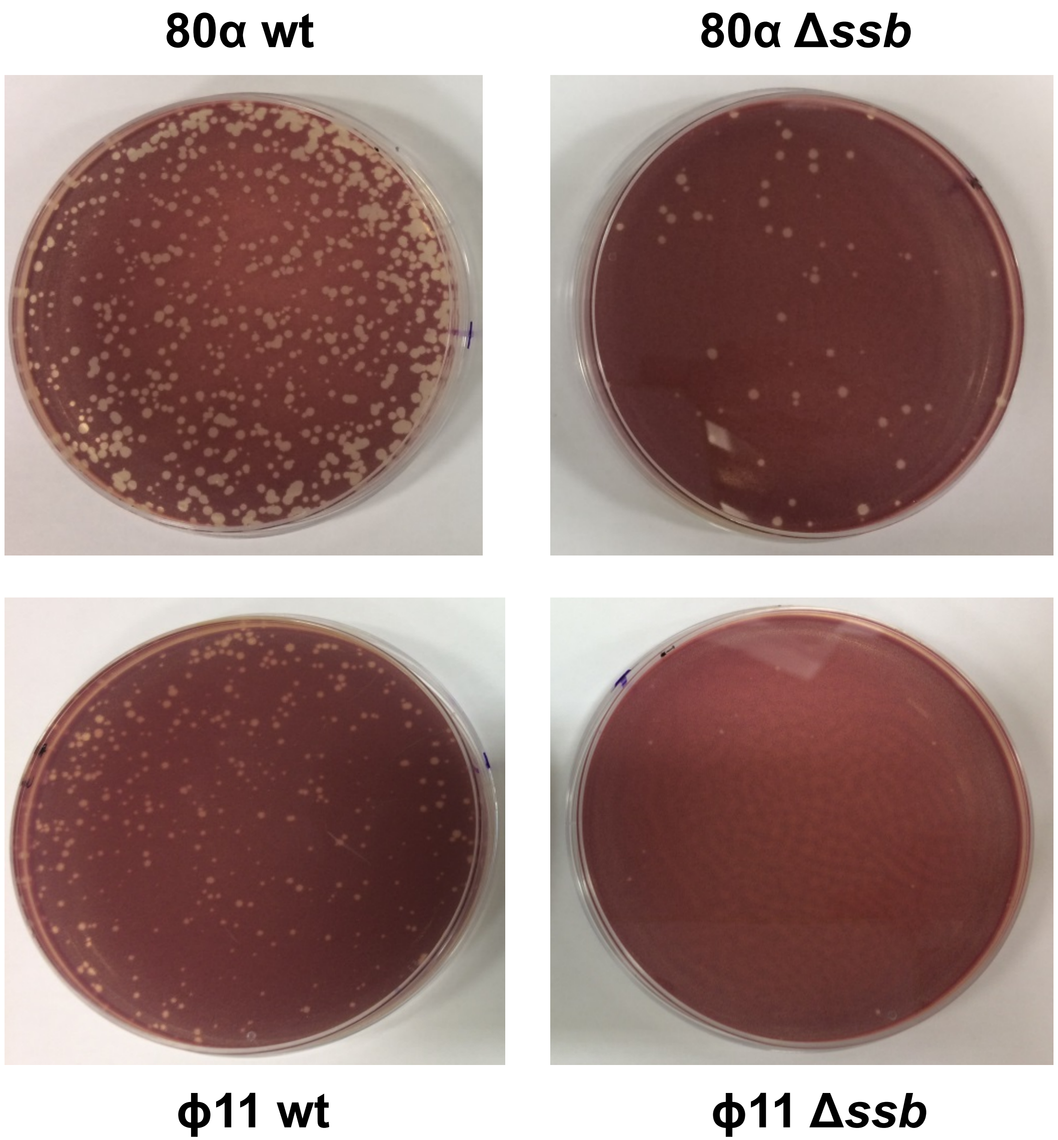

Figure S6. Effect of *ssb* mutation in phage plaque size. Approximately 10^8 bacteria (RN4220 strain) were infected with 400 p.f.u. of the corresponding phage, plated on phage bottom agar, and incubated 24h at 32 °C. Plates were stained with 0.1% TTC in TSB and photographed.

```
              1         10        20        30        40        50        60        70        80
80alpha_Sak   MTEQTLF..EQLNSKNVNDHTEQKNGLTYLAWSYAHQELKKIDPNYTVKVHEFPHPDINTENYFVPYLATPEGYFVQVSVTVKDSTETEW
11_Sak4       MTEKTNQDVDILTQLGVKDISKQNANKFYKFAIYGKF..GTGKTTFLTKDNNALVLDINEDGTT....VTEDGAVVQIKNYKHFSAVIKM

              90                   100          110         120       130                      140
80alpha_Sak   LPVL..DFRN..........KSLAKGSATTFD...INKAQKRCFVK..ASALHGLGLYIY...............NGEELPSASDNDI..
11_Sak4       LPKIIEQLRENGKQIDVVVIETIQKLRDITMDDIMDGKSKKPTFNDWGECATRIVSIYRYISKLQEHYQFHLAISGHEGINKDKDDEGST

                       150       160              170       180       190              200
80alpha_Sak   ......TELEERINQFVNLSQE.......KGRDATIDKTMRWLKISNINKLSQKQIAEAHQKLDAG.......LKQLDSEEK..Q
11_Sak4       INPTITIEAQDQIKKAVISQSDVLARMTIEEHEQDGEKTYQYVLNAEPSNLFETKIRHSSNIKINNKRFINPSINDVVQAIRNGN
```

Figure S7. Comparison of the 80α Sak and ϕ11 Sak4 recombinases. Identical residues are black boxed. Conserved residues are grey boxed.

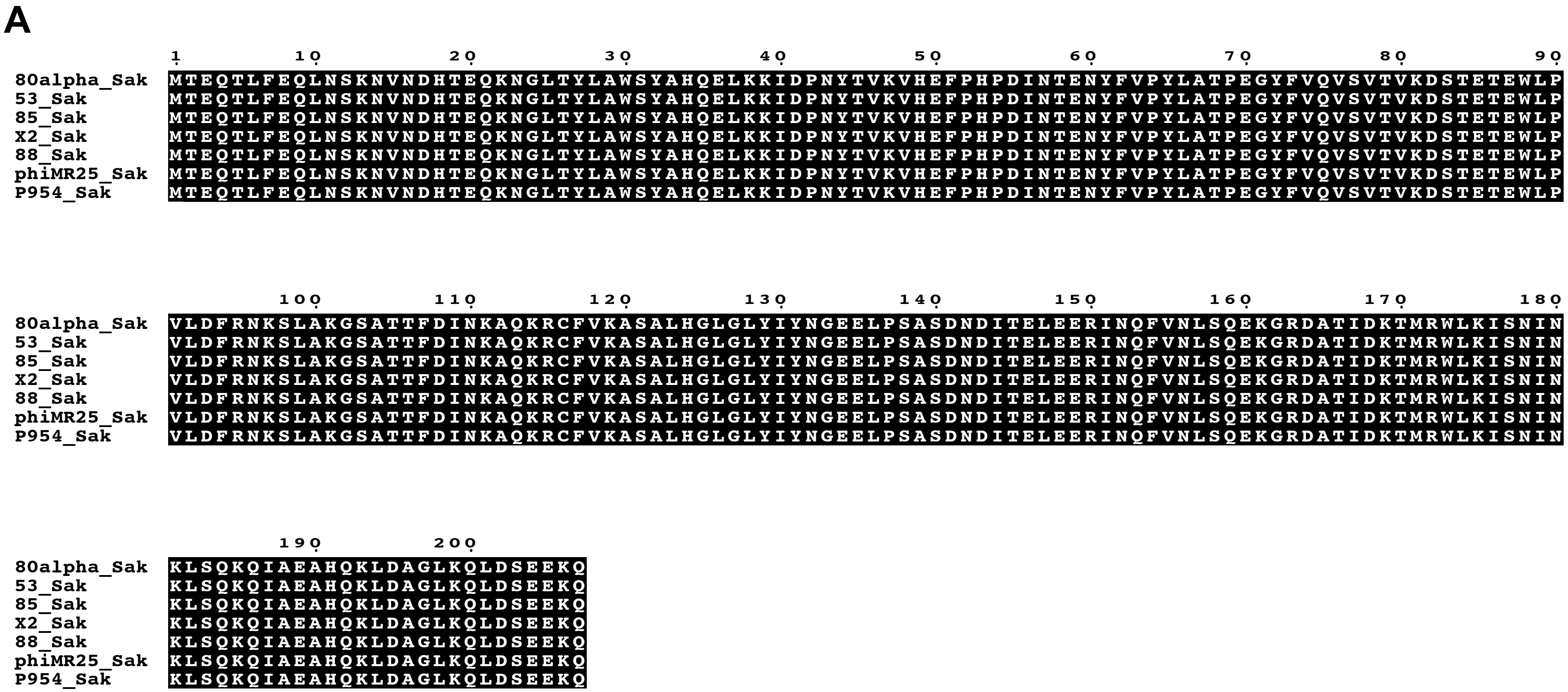

Figure S8A. Comparison of the Sak recombinases present in different *S. aureus* phages. Alignment was done with the M-coffee program and depicted with the ESPript server. Identical residues are black boxed

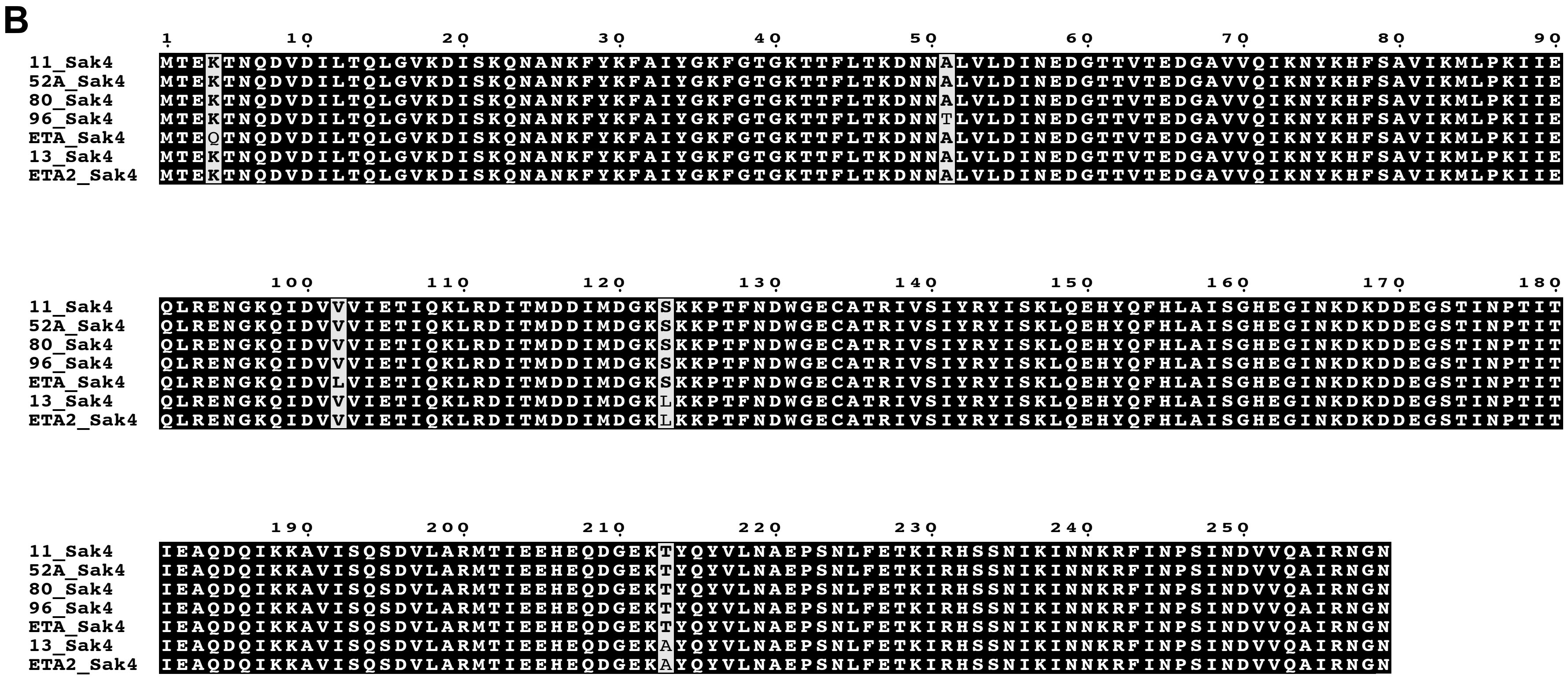

Figure S8B. Comparison of the Sak4 recombinases present in different *S. aureus* phages. Alignment was done with the M-coffee program and depicted with the ESPript server. Identical residues are black boxed. Conserved residues are grey boxed.

A

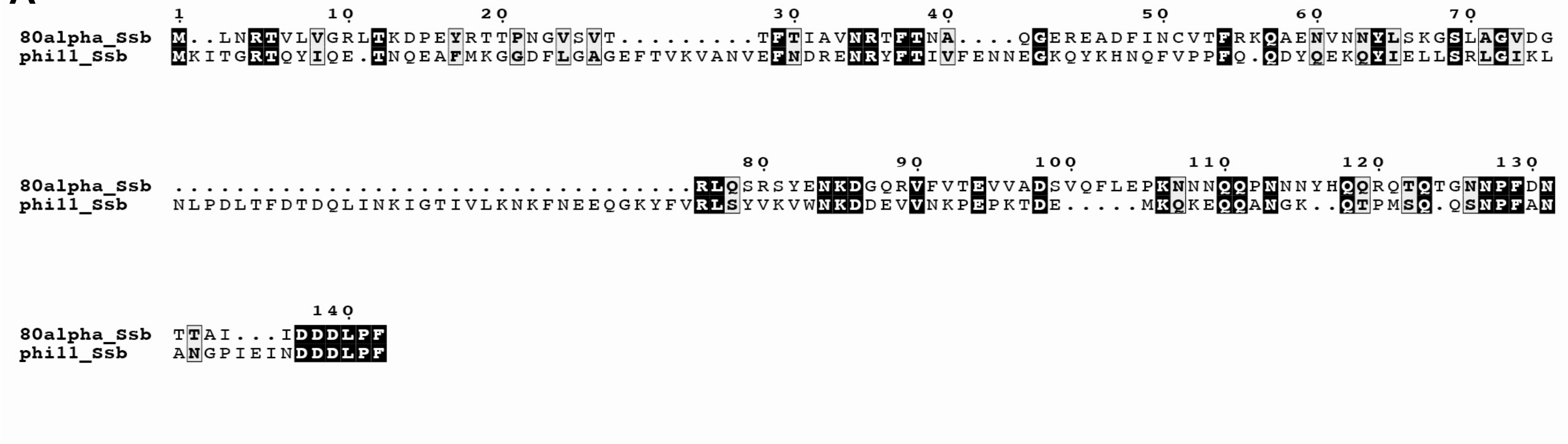

Figure S9A. Comparison of the 80α and ϕ11 Ssb proteins, as representative of the two different Ssb families. Alignment was done with the M-coffee program and depicted with the ESPript server. Identical residues are black boxed. Conserved residues are grey boxed.

B

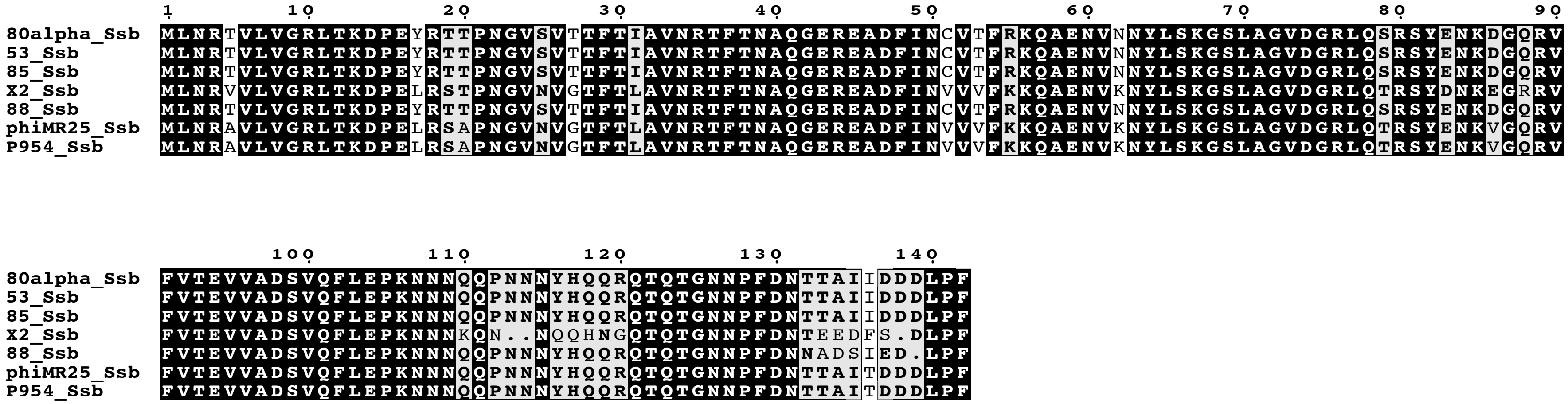

Figure S9B. Comparison of the Ssb proteins present in different *S. aureus* phages encoding Sak recombinases. Alignment was done with the M-coffee program and depicted with the ESPript server. Identical residues are black boxed. Conserved residues are grey boxed.

C

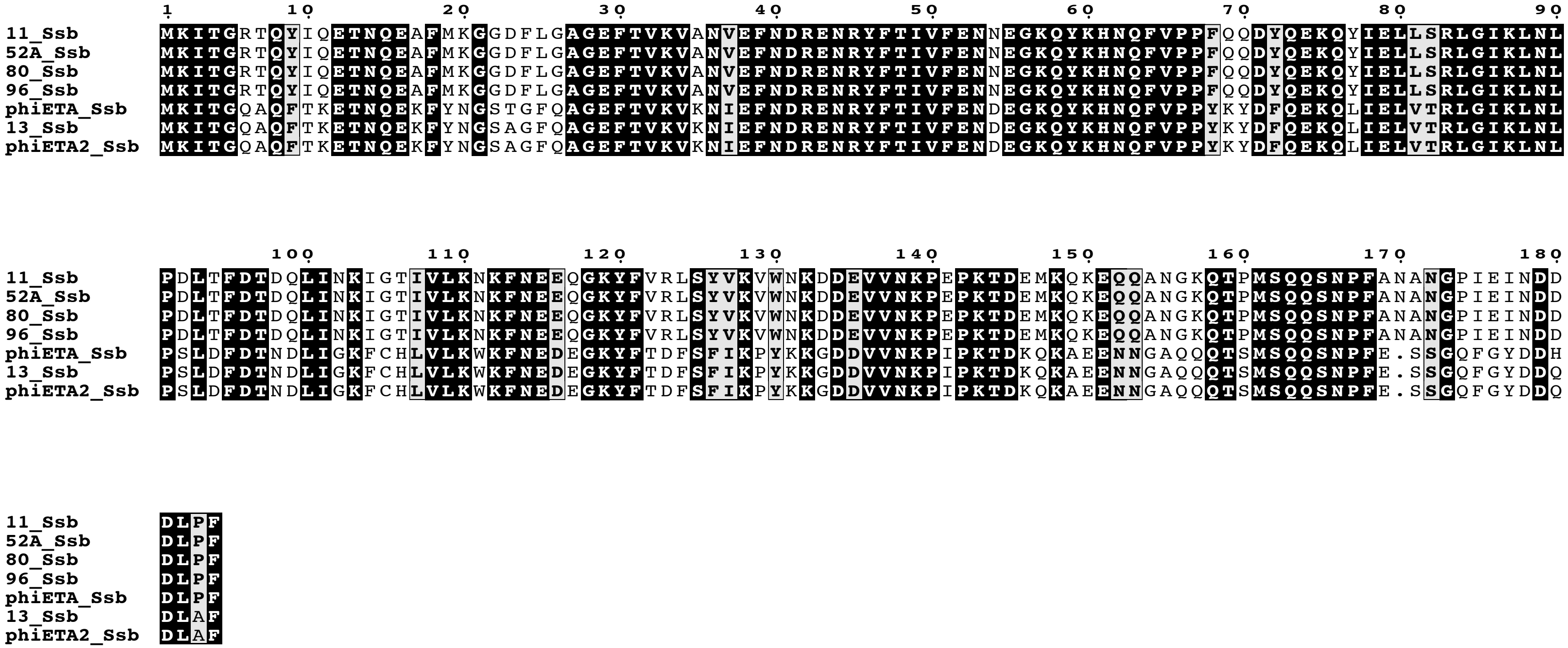

Figure S9C. Comparison of the Ssb proteins present in different *S. aureus* phages encoding Sak4 recombinases. Alignment was done with the M-coffee program and depicted with the ESPript server. Identical residues are black boxed. Conserved residues are grey boxed.
